# Transcriptional development of phospholipid and lipoprotein metabolism in different intestinal regions of Atlantic salmon (*Salmo salar*) fry

**DOI:** 10.1186/s12864-018-4651-8

**Published:** 2018-04-16

**Authors:** Yang Jin, Rolf Erik Olsen, Mari-Ann Østensen, Gareth Benjamin Gillard, Sven Arild Korsvoll, Nina Santi, Arne Bjørke Gjuvsland, Jon Olav Vik, Jacob Seilø Torgersen, Simen Rød Sandve, Yngvar Olsen

**Affiliations:** 1 Department of Biology, Faculty of Natural Sciences, Norwegian University of Science and Technology, NO-7491 Trondheim, Norway; 2 Centre for Integrative Genetics, Department of Animal and Aquacultural Sciences, Norwegian University of Life Sciences, Ås, Norway; 3 Department of Chemistry, Biotechnology and Food Science, Norwegian University of Life Sciences, Ås, Norway; 4 AquaGen AS, Postboks 1240, Sluppen, N-7462 Trondheim, Norway

**Keywords:** Atlantic salmon, Biosynthesis, Gene expression, Homologous genes, Intestinal regions, Lipoprotein, Phospholipids, RNA-Seq, Whole genome duplication

## Abstract

**Background:**

It has been suggested that the high phospholipid (PL) requirement in Atlantic salmon (*Salmo salar*) fry is due to insufficient intestinal *de-novo* synthesis causing low lipoprotein (LP) production and reduced transport capacity of dietary lipids. However, in-depth ontogenetic analysis of intestinal PL and LP synthesis with the development of salmon has yet to be performed. Therefore, in this paper we used RNA-Seq technology to investigate the expression of genes involved in PL synthesis and LP formation throughout early developmental stages and associate insufficient expression of synthesis pathways in salmon fry with its higher dietary PL requirement. There was a special focus on the understanding homologous genes, especially those from salmonid-specific fourth vertebrate whole-genome duplication (Ss4R), and their contribution to salmonid specific features of regulation of PL metabolic pathways. Salmon fry were sampled at 0.16 g (1 day before first-feeding), 2.5 and 10 g stages of development and transcriptomic analysis was applied separately on stomach, pyloric caeca and hindgut of the fish.

**Results:**

In general, we found up-regulated pathways involved in synthesis of phosphatidylcholine (PtdCho), phosphatidylethanolamine (PtdEtn), and LP in pyloric caeca of salmon between 0.16 and 10 g. Thirteen differentially expressed genes (*q* < 0.05) in these pathways were highly up-regulated in 2.5 g salmon compared to 0.16 g, while only five more differentially expressed (*q* < 0.05) genes were found when the fish grew up to 10 g. Different homologous genes were found dominating in stomach, pyloric caeca and hindgut. However, the expression of dominating genes in pathways of PL and LP synthesis were much higher in pyloric caeca than stomach and hindgut. Salmon-specific homologous genes (Ss4R) had similar expression during development, while other homologs had more diverged expression.

**Conclusions:**

The up-regulation of the *de-novo* PtdCho and PtdEtn pathways confirm that salmon have decreasing requirement for dietary PL as the fish develops. The similar expressions between Ss4R homologous genes suggest that the functional divergence of these genes was incomplete compared to homologs derived from other genome duplication. The results of the present study have provided new information on the molecular mechanisms of phospholipid synthesis and lipoprotein formation in fish.

**Electronic supplementary material:**

The online version of this article (10.1186/s12864-018-4651-8) contains supplementary material, which is available to authorized users.

## Background

Phospholipids (PL) are the main constituent of all biological cell membranes and separate the intracellular and extracellular aqueous environments. In addition to providing a structural scaffold for cell membranes, PL are also involved in numerous biological functions, such as provision of metabolic energy, cell membrane transport, and regulation of metabolism [1]. PL are also key structural components of lipoproteins (LP), which are involved in transport of dietary lipids from intestinal enterocytes to liver and peripheral tissues [2, 3]. It is well known that dietary inclusions of PL can improve growth and performance in many fish species including Atlantic salmon (*Salmo salar*) [4], Atlantic cod (*Gadus morhua*) [5], and rainbow trout (*Oncorhynchus mykiss*) [6]. Dietary and biliary PL is mainly digested by intestinal phospholipase A_2_ in fish, resulting in 1-acyl lyso-phospholipids (lyso-PL) and free fatty acids (FFA). Subsequently, both lyso-PL and FFA are absorbed into intestinal enterocytes and re-esterified into PL before being exported to the rest of the body [7]. In addition, PL can be synthesized *de-novo* in enterocytes from glycerol-3-phosphate (G-3-P) in fish [8].

A minimum requirement of PL is associated with early developmental stages of fish, but so far no minimum requirement has been demonstrated in adult fish [8]. In line with this, dietary PL has been shown to enhance growth and survival in Atlantic salmon fry up to 2.5 g, but not in larger fish [4, 9]. It has been suggested that the higher PL requirement is due to insufficient ability of *de-novo* synthesis in the intestine leading to low LP production and consequently reduced transport capacity of dietary lipids [8]. This was supported by previous histological studies in salmonids, showing lipid accumulation in intestinal enterocytes when fed PL deficient diet [9, 10]. However, these differences were not evident in a previous transcriptomic study where gene expressions in PL biosynthesis pathways were unchanged in 2.5 g salmon fed by PL-supplemented diet [11]. No study has examined the gene expression in salmon smaller than 2.5 g.

The intestinal tract of salmon consists of several regions, with different functions in lipid digestion, absorption and transport. It is generally believed that pyloric caeca (PC), rather than stomach (SM) or hindgut (HG), is the predominant region for lipid absorption and transport in salmon [12, 13]. Therefore, PL and LP were assumed to be mostly synthesized in PC region. However, other tissues like SM and HG could also have ability of synthesizing PL due to its structural roles in cell membranes [1]. LP has occasionally been observed in hindgut, suggesting some lipid absorption and transport activities in the region [14]. So far no study has demonstrated the expression of PL metabolic pathways in SM and HG of fish.

Many homologous genes in mammals were found belong to gene families controlling the same enzymatic processes but have distinct regulation in different tissues and developmental stages [1]. In this respect the Atlantic salmon has another layer of functional genome complexity as it experienced two extra rounds of whole genome duplication (WGD) compared to mammals, at the base of all teleost (Ts3R) and in a common ancestor of all salmonids ~ 100–80 Mya (Ss4R) [15]. Of the Ts3R and Ss4R gene duplicates, ~ 20 and 55% respectively are still retained as expressed genes in the genome [16]. This dramatic increase in the number of homologous genes in salmon thus necessitates a careful annotation of PL synthesis and LP formation pathway genes and their tissue-specific expression regulation to improve our understanding of salmon PL metabolism.

In this paper we annotate and characterize gene regulation involved in PL synthesis and LP formation in different intestinal regions (SM, PC and HG) during early developmental stages of salmon. Our aims are to (I) improve our understanding of the homologous genes, especially from Ss4R, and their contribution to salmonid specific features of regulation of PL metabolism pathways and to (II) specifically investigate the association of insufficient expression of PL and LP synthesis pathways in salmon fry with its higher dietary PL requirement.

## Methods

### Fish, diet and sampling procedure

Atlantic salmon eggs were hatched and cultivated at AquaGen Breeding Centre (Kyrksæterøra, Norway). From first-feeding, the fish were fed a normal commercial diet which satisfies the nutritional requirement of salmon, but without any additional PL supplement (Additional file 1). The diet was produced by EWOS AS (Bergen, Norway). Six salmon individuals were sampled at sizes of 0.16 g (*n* = 6, 1 day before first feeding), 2.5 g (*n* = 6, 65 days after first feeding) and 10 g (*n* = 6, 100 days after first feeding). The fish were euthanized by 1 g/L MS-222 (FINQUEL, Argent chemical labs, Washington, USA) buffered with same amount of sodium bicarbonate before dissection. The belly of the fish was dissected and immediately placed in 1 mL RNALater. Tissues were stored for 24 h at 4 °C for sufficient penetration of RNALater, before being dissected in sterile petri dish filled with RNALater. The entire intestinal tract was carefully separated from belly, connective tissues were removed and the intestinal contents were squeezed. Samples of stomach (SM, section between the end of esophagus and start of pyloric caeca), pyloric caeca (PC, section with fingerlike projections) and hindgut (HG, section after mid intestine) were dissected under dissecting microscope and immediately placed in in 2 mL cryo tubes (Thermo Fisher Scientific, Waltham, Massachusetts, USA) and transferred to − 80 °C before further analysis.

### RNA extraction, library preparation and transcriptome sequencing

The RNA extraction and library preparation were carried out in Centre for Integrative Genetics (CIGENE, Ås, Norway). Tissues from two individuals (*n* = 2 × 3) at 0.16 g were merged to obtain sufficient RNA for analysis. For individuals at 2.5 and 10 g, the RNA was extracted individually (*n* = 1 × 6). Total RNA was extracted from SM, PC and HG using RNeasy Plus Universal Kits (QIAGEN, Hilden, Germany), according to manufacturer’s instruction. RNA concentration and purity were assessed by Nanodrop 8000 (Thermo Scientific, Wilmington, USA). RNA integrity was checked by Agilent 2100 Bioanalyzer (Agilent Technologies, Santa Clara, CA, USA). All samples had a RIN value > 8, sufficient for transcriptome analysis. RNA libraries were prepared by using TruSeq Stranded mRNA Library Prep Kit (Illumina, San Diego, CA, USA), according to manufacturer’s instruction. Samples were sequenced using 100 bp single-end high-throughput mRNA sequencing (RNA-Seq) on Illumina Hiseq 2500 (Illumina, San Diego, CA, USA) in Norwegian Sequencing Centre (Oslo, Norway).

### Identification of genes and phylogenetic analysis

The genes involved in PL synthesis and LP formation in Atlantic salmon (*Salmo salar*) were manually annotated by matching salmon to zebrafish (*Danio rerio*) orthologs from the KEGG reference pathway of glycerolphospholipid metabolism and other studies [17–19]. Ortholog group predictions were carried out using Orthofinder (v0.2.8) on proteins from eight fish species: zebrafish (*Danio rerio*), stickleback (*Gasterosteus aculeatus*), medaka (*Oryzias latipes*), Northern Pike (*Esox lucius*), grayling (*Thymallus thymallus*), rainbow trout (*Oncorhynchus mykiss*), coho salmon (*Oncorhynchus kisutch*), Atlantic salmon (*Salmo salar*), and two mammal outgroup species: human (*Homo sapiens*) and mouse (*Mus musculus*). The protein sequences within orthogroups were aligned to each other using MAFTT and maximum likelihood trees were estimated using FastTree. Orthogroup trees were subsequently split into smaller clan trees using an in-house R script (clanfinder.R, available from https://github.com/srsand/Phylogenomics/blob/master/clanfinder.R). For zebrafish proteins in selected KEGG reference pathways, salmon proteins within the same protein clan tree were annotated using the zebrafish KEGG Orthology terms. The detailed information on Orthogroup prediction and phylogenetic analysis was published elsewhere [18]. All annotated salmon genes were grouped into a PL gene list and used for gene expression analysis (Additional file 2).

### RNA-sequencing data and statistical analysis

Read sequences were quality trimmed, removing any Illumina TruSeq adapter sequence and low quality bases (Phred score < 20) from read ends and length filtered (minimum length 40 bases) using cutadapt (v1.8.1), before being aligned to the salmon genome (ICSASG_v2) using STAR (v2.5.2a). Raw gene counts per sample were generated from read alignments using HTSeq-count (v0.6.1p1) and the NCBI salmon genome annotation (available for download at http://salmobase.org/Downloads/Salmo_salar-annotation.gff3.gz). The uniquely mapped reads, aligned to exon regions, were counted for each gene in the annotation.

For each tissue type (SM, PC, HG), a differential expression analysis (DEA) was performed comparing all genes in 2.5 g and 10 g samples to the 0.16 g samples. Genes were filtered prior to DEA testing by a minimum count level of at least 1 count per million (CPM) in two or more samples, to remove genes with too few counts for testing. From raw counts, DEA was conducted using R package edgeR (v3.8.6) with pairwise exact tests to produce gene fold changes and false discovery rate (FDR) adjusted *p* values (*q*). Genes with *q* < 0.05 were considered to be differentially expressed genes (DEGs) between two test conditions. A KEGG ontology enrichment analysis (KOEA) was also conducted using edgeR. For KO terms, *p* values were generated based on the number of DEGs compared to the total number of genes annotated to each KO term. The PL gene list was used to subset the total DEA results to find DEGs involved in the synthesis of PL and LP.

To visually compare expression levels between different genes and tissues, normalized counts in the form of transcripts per million (TPM) values were generated. Raw gene counts were first divided by their mRNA length in kilobases to normalize for transcript length, then divided by the total number of counts from each library to normalize for sequencing depth. Sample library sizes were normalized to each other using the edgeR TMM normalization method.

All RNA-Seq data analysis was preformed using R (v3.2.4) and Bioconductor (v3.3). The pathway maps of PL and LP synthesis were produced using PathVisio. The heatmap was drawn using R package pheatmap. All other figures were produced using SigmaPlot for Windows Version 13.0.

## Results

### Annotation of PL pathway genes in salmon

To examine expression of genes involved in PL and LP synthesis in salmon, we created a list of all PL-related salmon genes in the pathways based on their zebrafish orthologs identified in previous studies. A total of 62 zebrafish genes involved in PL *de-novo* synthesis, lyso-PL synthesis, PL turnover and LP synthesis pathways were used to identify PL metabolism genes in salmon. In total, 125 corresponding salmon homologs were identified based on their phylogenetic relationship to human and zebrafish orthologs. For each zebrafish gene, 1 or more salmon orthologs were identified. Due to the Ss4R WGD, 67% of the PL genes contain a salmon-specific duplicate in the genome. Homologous genes involved in the same enzymatic reaction were grouped into a family for comparison of gene expression. A summary of identification and nomenclature of PL genes is shown in Additional file 2.

### Tissue specific regulation of PL metabolism in the gut

An average total of 22 million reads were sequenced from each library, out of which ~ 85% were mapped to the salmon genome ICSASG_v2 (Additional file 3). From a total of 81,574 genes currently annotated in the salmon genome, 31,411 genes passed a minimum level of read counts for use in DEA. DEA was carried out on SM, PC, and HG separately to assess the extent of developmentally associated changes by comparing 2.5 g and 10 g salmon to 0.16 g (Additional file 4). The different intestinal regions differed greatly in the number of differentially expressed genes (DEGs, *q* < 0.05), with 10% of genes differentially expressed in SM, and around 30% in PC and HG (Table 1). A KEGG ontology enrichment analysis (KOEA) on DEGs in SM, PC, and HG of salmon identified pathways which were significantly (*p* < 0.05) regulated between different stages of development (Additional file 5). This included the glycerophospholipid metabolism pathway, which was significantly regulated in PC and HG, but not SM.

**Table 1:** Number of significantly (q < 0.05) differentially expressed genes (DEGs) between 2.5, 10 and 0.16 g salmon

	DEGs	Percentage of total genes
All genes		
2.5 g vs 0.16 g SM	3369	11%
10 g vs 0.16 g SM	2897	9%
2.5 g vs 0.16 g PC	8552	27%
10 g vs 0.16 g PC	11,427	36%
2.5 g vs 0.16 g HG	9052	29%
10 g vs 0.16 g HG	10,475	33%
PL genes		
2.5 g vs 0.16 g SM	22	18%
10 g vs 0.16 g SM	10	8%
2.5 g vs 0.16 g PC	47	40%
10 g vs 0.16 g PC	64	52%
2.5 g vs 0.16 g HG	45	36%
10 g vs 0.16 g HG	49	40%

The total number of genes was 31,411, with 124 genes involved in PL biosynthesis and LP formation pathways (PL genes)

**Fig. 1:**
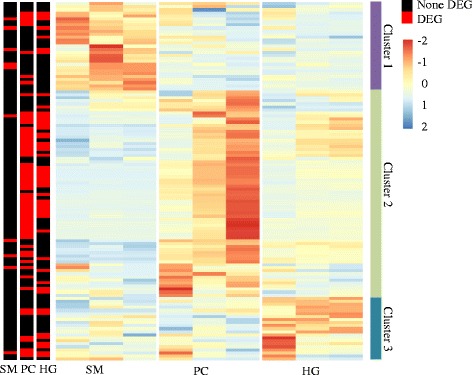
Expression of genes in phospholipid and lipoprotein synthesis pathways between different intestinal regions of salmon. For each tissue, the three columns represent 0.16 g, 2.5 g and 10 g samples from left to right. The color intensity is relative to the standard deviation from mean of TPM over developmental stages and tissues (row-scaled). Differential expressed genes (DEG, *q* < 0.05) between 0.16 g, 2.5 g and 10 g samples were annotated in three columns, which represent stomach (SM), pyloric caeca (PC) and hindgut (HG) respectively from left to right

The relative expression of genes involved in PL synthesis and LP formation showed categorization into three distinct tissue related clusters associated with developmental differences (Fig. 1). The genes in cluster 2 were characterized by having highest expression in PC, while the remaining genes were either highest expressed in SM (cluster 1) or HG (cluster 3). Only a few genes were down-regulated during the development of fish, whereas most genes showed onset of expression, especially in cluster 2. The differentially expressed PL genes were annotated in Fig. 1. Similar to genome-wide changes in expression, PL genes PC and HG were much more responsive (40% DEGs) to development compared to SM (less than 20% DEGs). Most DEGs were shared between PC and HG in cluster 2, while fewer DEGs were in other clusters. Moreover, the shared DEGs showed a much larger change in PC than in HG during development, resulting in increasing difference of expression between PC and HG as salmon grew.

### Regulatory divergence of homologous genes among developmental stages and intestinal sections among Ss4R duplicates

**Fig. 2:**
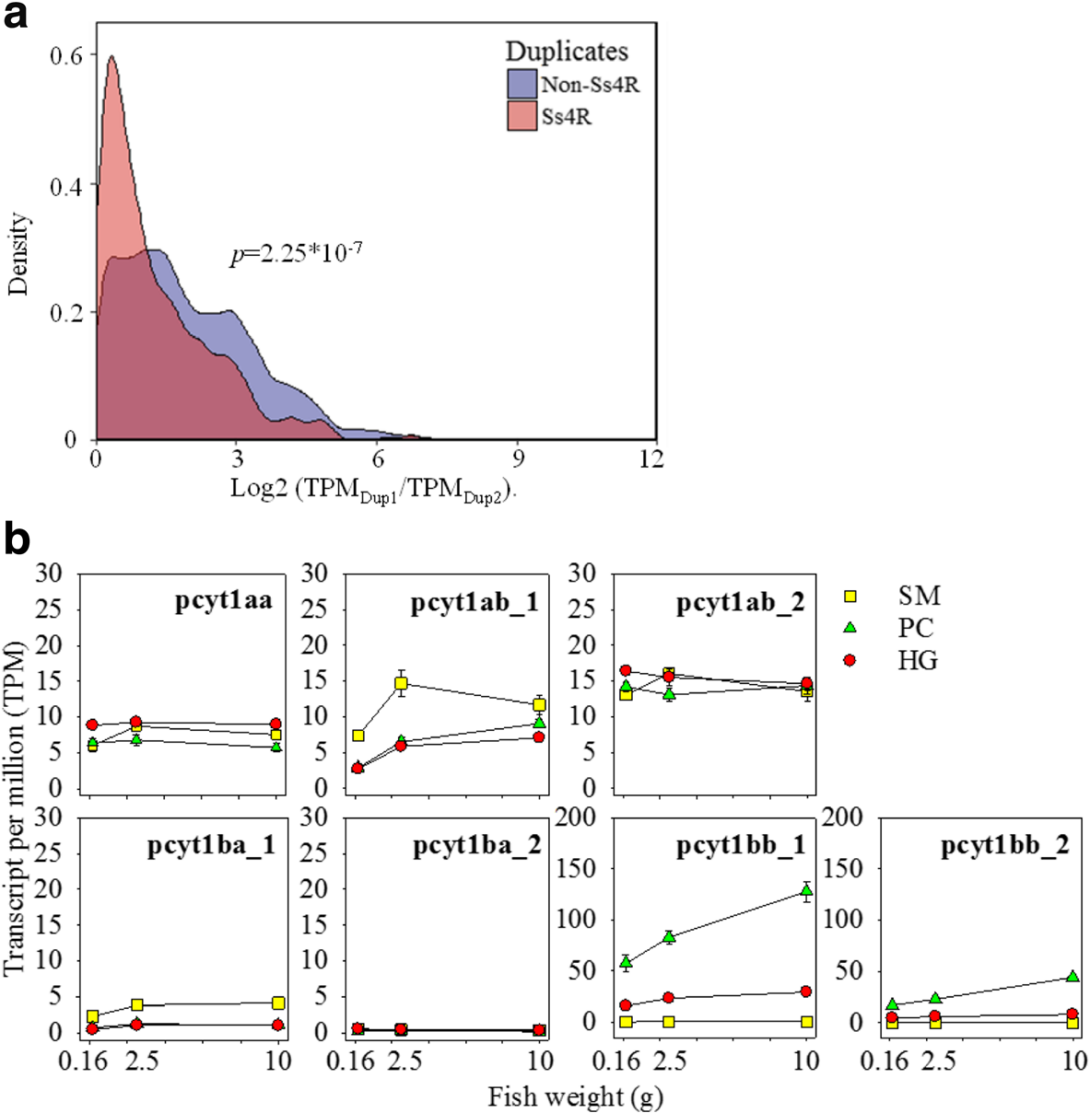
Comparison of gene duplicates over developmental stages and intestinal regions. **a** The distribution of expressional differences between of genes in each homologous gene family. Gene expression in transcripts per million (TPM) was compared between pairs of every gene homolog, by taking the ratio of the higher expressed gene (TPM_Dup1_) over the lower expressed (TPM_Dup2_). Expression ratios of gene pairs were calculated for each tissue and development stage. The density of expression ratios on a log2 scale is showed for Ss4R and non-Ss4R homolog gene pairs. A t-test between mean expression ratios of Ss4R and non-Ss4R showed a significant (*p* = 2.25 × 10^− 7^) difference. **b** Comparison of *pcyt1* genes in TPM over developmental stages and intestinal regions. The gene expressions were compared over three developmental stages (0.16, 2.5 and 10 g) in stomach (SM), pyloric caeca (PC) and hindgut (HG) of salmon. Numbers after underline indicates Ss4R gene duplicates specific in salmonids

The relative expression (TPM value) of all genes in the PL and LP synthesis pathways are summarized in Additional file 6. The salmon-specific (Ss4R) duplicate pairs of genes showed a significantly (*p* < 0.05) more similar expression than other duplicate pairs in each homologous gene family, regardless of developmental stages and intestinal sections (Fig. 2a). Moreover, the highest expression levels of the homologous genes were mostly found in PC rather than in SM or HG, which suggests that PC is the most important intestinal section for PL synthesis and LP formation in salmon (Additional file 6).

*Pcyt1* family is a representative example of the regulatory complexity of homologous genes among intestinal sections and developmental stages (Fig. 2b). The *pcyt1* family has 2 members in mammals (*pcyt1a* and *pcyt1b*), 4 members in zebrafish (*pcyt1aa*, *pcyt1ab*, *pcyt1ba*, and *pcyt1bb*) and 7 members in salmon (*pcyt1aa*, *pcyt1ab_1*, *pcyt1ab_2*, *pcyt1ba_1*, *pcyt1ba_2*, *pcyt1bb_1*, and *pcyt1bb_2*). Among all homologous genes, *pcyt1bb_1* had much higher expression levels in PC than in SM and HG. It was also the most highly expressed homolog in all tissues. The expression level of *pcyt1bb_1* in PC and HG both more than doubled as the fish grew from 0.16 g to 10 g. The Ss4R homologous genes, *pcyt1bb_1* and *pcyt1bb_2*, showed similar divergence in expression levels between tissues and developmental stages. The expressions of *pcyt1ab_1* and *pcyt1ba_1* genes were both higher in SM than PC and HG, while *pcyt1aa*, *pcyt1ab_2*, and *pcyt1ba_2* were similarly expressed between the three tissues.

### Regulation of PL synthesis and LP formation pathways in PC

**Fig. 3:**
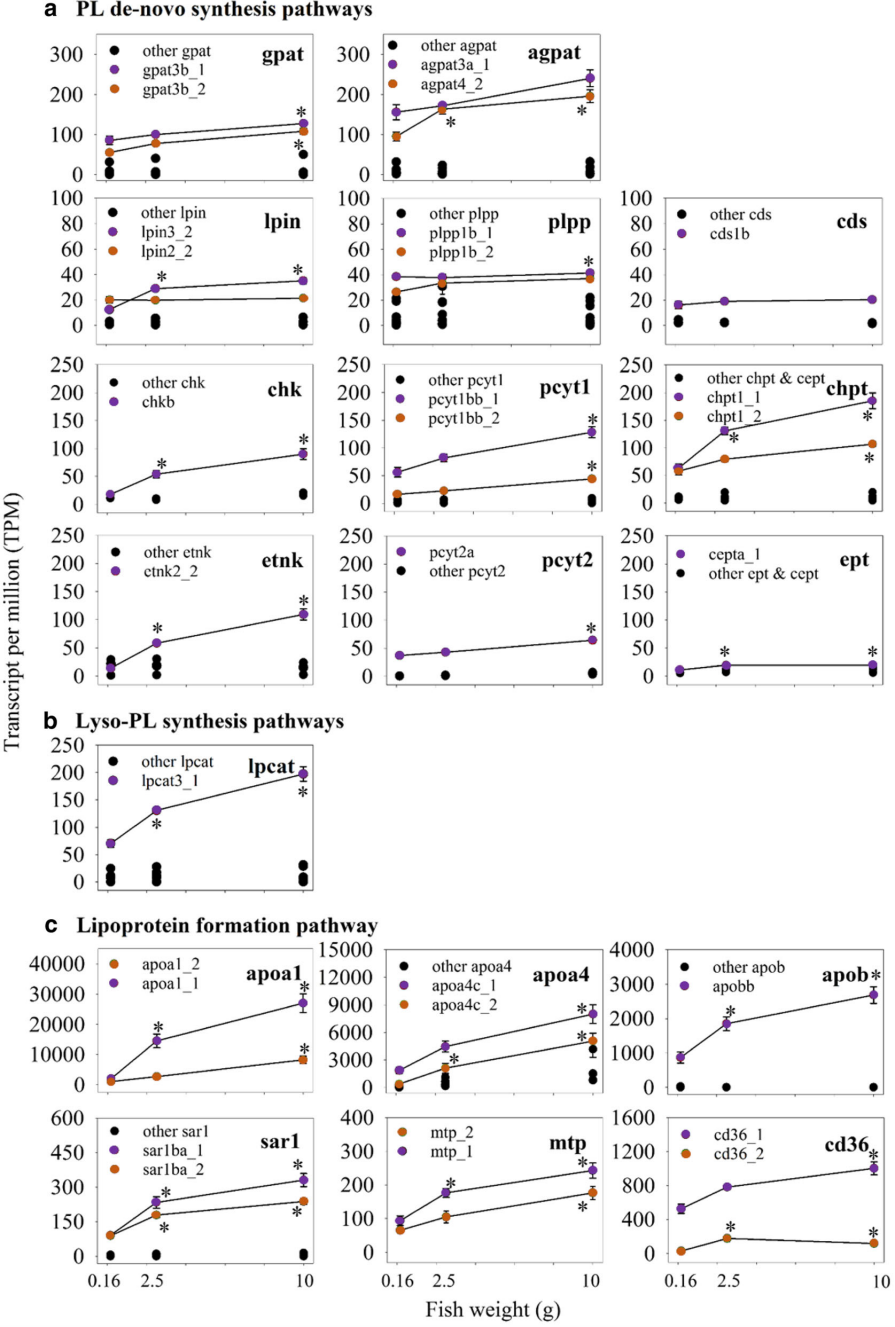
Expressions of key genes in phospholipid and lipoprotein synthesis pathways in pyloric caeca at early stage of salmon. Eighteen families of homologous genes in phospholipid (PL) *de-novo* synthesis (**a**), lyso-phospholipid (lyso-PL) synthesis (**b**) and lipoprotein formation (**c**) pathways are shown for comparing their relative expression in transcripts per million (TPM) between 0.16, 2.5 and 10 g fish. Genes with high TPM are marked in purple and orange, while other genes were all marked in black. Points that significantly different in expression (*q* < 0.05) compared to 0.16 g using differential expression analysis on raw counts are annotated with an asterisk

It clear from our expression analyses (Fig. 1) that PC is the most transcriptionally active tissue, measured as expression levels of genes involved in PL and LP metabolic pathways. Therefore, PC was selected for a detailed study of the differences in expression of homologous genes between 0.16 g, 2.5 g, and 10 g salmon (Fig. 3). The homologous genes in 18 families of key genes in PtdCho, PtdEtn and LP synthesis pathways were thus selected for in depth analyses.

In most gene families, one or two genes had much higher expression than their homologs, with all being up-regulated during development (Fig. 3). In the 11 families with two highly expressed genes, they are mostly salmon-specific duplicates from Ss4R WGD. In *de-novo* synthesis pathways, highly expressed genes were mostly significantly (*q* < 0.05) up-regulated from 0.16 g to 10 g (Fig. 3a). The highly expressed genes involved in the PtdCho synthesis pathway (*chk*, *pcyt1*, and *chpt* families) showed a much more pronounced increase compared to genes in other *de-novo* synthesis pathways. When we compared TPM of the highly expressed genes, *lpin* and *plpp* had slightly higher expression compared to *cds* family. Genes in *ept* family had much lower expression than other families in the PL de novo synthesis pathway. In lyso-PL synthesis pathways, the expression of *lpcat3a* was largely increased (*q* < 0.05) during development, whereas other *lpcat* genes remained stable (*q* > 0.05, Fig. 3b). *Lpgat1a* and *lpiat1* in other families in lyso-PL pathways were also up-regulated (*q* < 0.05) during development (Additional file 7). All other genes in lyso-PL synthesis pathways were expressed at much lower levels than *lpcat3a*. Genes in LP formation pathway had much higher expression than in PL synthesis pathways (Fig. 3c). All highly expressed genes in LP formation pathway were largely up-regulated (*q* < 0.05) between 0.16 and 10 g salmon.

**Fig. 4:**
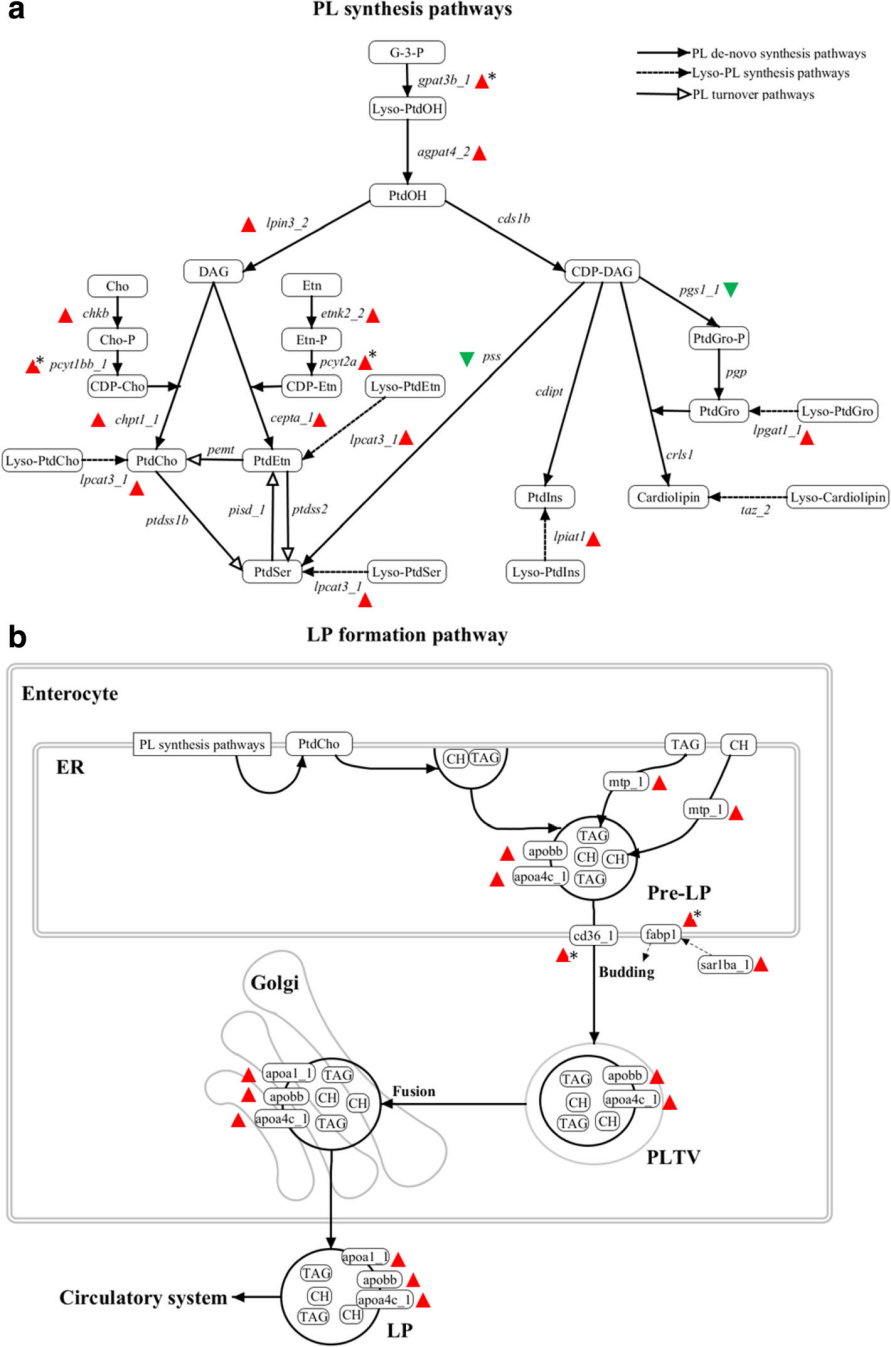
Comparison of phospholipid (PL) synthesis and lipoprotein (LP) formation pathways between 0.16, 2.5 and 10 g salmon. Colored triangles indicate the significantly (*q* < 0.05) up (red) or down (green) regulation of the highest expressed genes found in each enzymatic step of the pathways. Asterix indicates genes only significantly (*q* < 0.05) changed between 0.16 g and 10 g. **a** PL *de-novo* synthesis, lyso-PL synthesis and PL turnover pathways in fish. Glycerol-3-phosphate (G-3-P) is first acylated by acyltransferases to phosphatidic acid (PtdOH), which can be transferred into diacylglycerol (DAG) or CDP-diacylglycerol (CDP-DAG) by phosphatidate phosphatase (plpp and lpin) or CDP-DAG synthetase (cds). DAG is utilized with CDP-choline (CDP-Cho) and CDP-ethanolamine (CDP-Etn) for synthesizing of phosphatidylcholine (PtdCho) and phosphatidylethanolamine (PtdEtn). CDP-DAG is utilized for synthesizing of phosphatidylserine (PtdSer), phosphatidylglycerol (PtdGro), phosphatidylinositol (PtdIns) and Cardiolipin. **b** LP formation pathway in enterocyte of fish. PtdCho is synthesized on the membrane of endoplasmic reticulum (ER) through *de-novo* synthesis, turnover or lyso-PL pathway before used for pre-lipoprotein (Pre-LP) formation. Pre-LP is a nascent lipoprotein assembled by PtdCho, triacylglycerol (TAG), cholesterol (CH), apolipoprotein B (apob) and apolipoprotein AIV (apoa4). Pre-LP is then targeted to the Golgi apparatus via pre-lipoprotein transport vesicle (PLTV) generated by ER. The maturation of Pre-LP happens in Golgi, where apolipoprotein AI (apoa1) is added before secreting into circulatory system

The proposed transcriptional regulation of PL synthesis and LP formation pathways is summarized in Fig. 4. By summarizing the change of the highly expressed genes in PC, we found up-regulation of genes in the *de-novo* PtdCho and PtdEtn synthesis pathways and down-regulation of genes in the *de-novo* PtdSer and PtdGro synthesis pathways between 0.16 and 10 g salmon (Fig. 4a). Other *de-novo* synthesis pathways were not changed during development. The Lyso-cardiolipin synthesis pathway was not changed, while other lyso-PL synthesis pathways were all up-regulated. Expression of genes in the phospholipid turnover pathways were not changed during development. The LP formation pathway was up-regulated between 0.16 and 10 g (Fig. 4b).

## Discussion

The objective of the present study was to explore the transcriptional changes of PL synthesis and LP formation pathways in different intestinal regions of salmon during early developmental stages. By integrating RNA-Seq data with a manually curated automated sequence ortholog prediction, we identified many DEGs in SM, PC, and HG during development of salmon. Most of those changes occurred between the onset of feeding at 0.16 and 2.5 g, and with additional changes between 0.16 and 10 g. This shows that the maturation of lipid metabolic pathways progressed for a substantial period following the completion of yolk sac reabsorption. By comparing the highly expressed genes in each family, we found a continuous increased capacity of PtdCho, PtdEtn, and LP synthesis in PC from 0.16 to 10 g. This implies an increased capacity for PL and LP synthesis after onset of feeding.

Our results are in line with previous qPCR-based targeted gene studies in salmon in which several major PL biosynthetic genes were up-regulated in PtdCho and PtdEtn synthesis between 2.5 and 10 g [20]. However, we did not observe a clear leveling-off of expression levels of synthesis genes at 10 g, suggesting that the completion of intestinal maturation might take longer time to accomplish. This supports the hypotheses of a higher dietary PL requirement in salmon fry compared to larger fish [10].

The present study was the first to investigate expression differences between homologous genes in PL and LP synthesis pathways in salmon. Several homologous genes were identified that encode enzymes that catalyze the same reaction in PL and LP synthesis. In most cases, we found that one or two genes had much higher expression levels than their homologs for a given tissue, suggesting these genes to be key regulators in the pathways. However, the expression levels of the homologous genes appear to vary with both intestinal regions and developmental stages. This resulted in different genes dominating in expression levels in SM, PC and HG. The differences in expression are likely due to differences in function, subcellular localization, and developmental stages of homologous genes in the tissues [1]. More interestingly, the salmon-specific homologous genes derived from Ss4R WGD were expressed at similar levels in different tissues and developmental stages, while other homologous genes seemed to have more differences in expression. This may suggest a functional divergence of Ss4R homologous genes was incomplete compared to homologs from other genome duplications. This supports the recent study in which 55% of the Ss4R homologous genes were found to have similar expression levels among 15 tissues in salmon [16].

The present study was the first to utilize RNA-Seq to investigate differences in global gene expression patterns among different intestinal regions in PL metabolism. PC is the predominant region for lipid absorption and transport in salmon [12, 13]. This is consistent with higher expression of genes involved in PtdCho, PtdEtn, and LP synthesis pathways in PC rather than SM and HG. As PtdCho is the predominant lipid class forming the membrane fraction of LP, the higher expression of genes in PtdCho synthesis pathway in PC suggests a high rate of LP production [3, 8]. This has been confirmed by histological observation of large lipid droplets accumulating in midgut enterocytes of PL-deficient fish, while little droplets were found in fish fed dietary PtdCho [9, 10]. The expression of genes involved in PL synthesis in SM and HG were most likely related to other biological functions such as cell maintenance and metabolism. Almost no expression of LP formation genes was found in SM, in agreement with the general observation that SM is not involved in lipid digestion, uptake, and transport. However, despite being expressed at low levels, many genes of the LP formation pathways were found in HG, suggesting some capacity to absorb and transport lipid in this intestinal region [14].

Several highly expressed genes in PL and LP synthesis pathways of salmon do not conform to the known function and regulation of these pathways in mammals. For example, it is believed that choline kinase (CK) α enzyme is critical for PtdCho maintenance in most tissues in mammals, whereas CKß enzyme is only essential in muscle tissue [21, 22]. In salmon, the expression of the *chkb* gene, encoding for CKß, was significantly elevated in PC after start feeding, whereas *chka* genes were unchanged. Similarly, the expressions of *pcyt1a* genes were relatively low in PC during early development, while *pcyt1bb_1* notably increased in PC after onset of feeding. Therefore, we assume that the production of PtdCho for LP synthesis is probably through a compensatory pathway controlled by *chkb* and *pcty1b*-encoded enzymes and activated in PC after switching to external feeding. On the other hand, there may be another pathway controlled by *chka* and *pcyt1a*-encoded enzymes, which produce PtdCho to maintain cell growth and survival. This suggestion agrees with previous studies pointing to the subcellular location of the enzymes [23, 24]. The CTP: phosphocholine cytidylyltransferase (CCT) α, which is the product of the *pcyt1a* gene, is predominantly located in the nucleus. On the other hand, *pcyt1b*-encoded CCTß is localized in the endoplasmic reticulum (ER) and the cytosol, which could be utilized in synthesizing PtdCho for LP formation. However, as the level of gene expression does not always directly reflect relative importance of two similar enzymes in a pathway, the posttranslational modification like phosphorylation of CCT could also be critical in regulating the activity of enzymes without affecting the mRNA level [1].

## Conclusions

The present study has provided new information on transcriptional events that may regulate PL synthesis and LP formation in salmon fry. By comparing the expression levels of homologous genes, we identified several genes which had highly expression among their homologs in PtdCho, PtdEtn, and LP synthetic pathways in PC of salmon. Those highly expressed genes were all up-regulated during development, suggesting the increasing capacity for PL synthesis and LP formation. Given the lower expression of PL genes in the early life of salmon and the link to PL requirement for growth, it seems likely that the critical supplementation point is during the onset of feeding, then decreases as the fish grows. The expression levels of the homologous genes in PL synthesis and LP formation pathways appeared to vary with both intestinal regions and developmental stages. This resulted in different transcripts dominating in abundance in SM, PC and HG. The salmon-specific homologous genes derived from Ss4R WGD were expressed similarly among different tissues and developmental stages, while homologous genes from other genome duplications seemed to have a different expression pattern. More studies on both gene expression and protein abundance are required to confirm these relationships during early stages of salmon development. Considering the present results on identification of key regulating genes in PL synthesis and lipid transport, we suggest a future study on the dietary requirement of PL at first-feeding stage, which focuses on the changes of key regulating genes involved in PL and LP synthesis pathways in PC of salmon.

## Additional files

Additional file 1: **Table S1.** Composition and nutritional value of the diet used in current experiment. (DOCX 15 kb)

Additional file 2: **Table S1.** List of Atlantic salmon (Ssa) genes involved in phospholipid (PL) *de-novo* synthesis, lyso-PL synthesis, and lipoprotein (LP) formation pathways. Nomenclature of salmon genes was based on their human (Hsa) and zebrafish (Dre) orthologs. Numbers after underline in Ssa names indicate salmon-specific gene duplicates. NCBI gene ID of salmon and zebrafish is also listed in table. Reference listed the origin of zebrafish genes used for identification of salmon genes. (DOCX 29 kb)

Additional file 3: **Table S1.** Summary of mapping statistics of all 45 samples used for RNA sequencing (2 fish each replicate × 3 replicates × 3 tissue in 0.16 g fish, 1 fish each replicate × 6 replicates × 3 tissue in 2.5 and 10 g fish). (DOCX 16 kb)

Additional file 4: **Table S1.** Result of differential expression ananlysis on all genes in stomach, pyloric caeca and hindgut of 2.5 and 10 g salmon both compared to 0.16 g. (XLSX 14268 kb)

Additional file 5: **Table S1.** Result of KEGG ontology enrichment analysis on differential expressed genes (DEG) in stomach, pyloric caeca and hindgut of 0.16 and 2.5 g salmon both compared to 0.16 g. (XLSX 65 kb)

Additional file 6: **Table S1.** Transcript per million (TPM) of all gene duplicates in phospholipid and lipoprotein synthesis pathways in stomach, pyloric caeca, and Hindgut of 0.16, 2.5 and 10 g salmon. (DOCX 32 kb)

Additional file 7: **Table S1.** Log2 fold change (LogFC) and adjusted *p* value (*q*) of all gene duplicates in phospholipid and lipoprotein synthesis pathways in stomach, pyloric caeca and hindgut of 2.5 and 10 g salmon both compared to 0.16 g. (DOCX 34 kb)

## Abbreviations

agpat: 1-acylglycerol-3-phosphate acyltransferases
cd36: cluster of differentiation 36
cdipt: cdp-diacylglycerol-inositol 3-phosphatidyltransferase
CDP: Cytidine diphosphate
CDP-Cho: CDP-choline
CDP-DAG: CDP-diacylglycerol
CDP-Etn: CDP-ethanolamine
cds: CDP-DAG synthase
cept: CDP-choline/ethanolamine: diacylglycerol phosphotransferase
CH: Cholesterol
chk: choline kinase
Cho-P: Phosphocholine
chpt: CDP-choline: diacylglycerol phosphotransferase
crls: Cardiolipin synthase
DAG: Diacylglycerol
ept: CDP-ethanolamine: diacylglycerol phosphotransferase
ER: Endoplasmic reticulum
etnk: ethanolamine kinase
Etn-P: Phosphoethanolamine
fabpl: Fatty acid binding protein, liver
G-3-P: Glycerol-3-phosphate
gpat: glycerol-3-phosphate acyltransferase
lclat: lysocardiolipin acyltransferase
lpcat: lysophosphatidylcholine acyltransferase
mboat: membrane bound o-acyltransferase
mtp: microsomal triglyceride transfer protein
pcyt1: choline-phosphate cytidylyltransferase
pcyt2: Ethanolamine-phosphate cytidylyltransferase
pemt: phosphatidylethanolamine methyltransferase
pgp: phosphatidylglycero phosphatase
pgs: CDP-diacylglycerol: glycerol-3-phosphate phosphatidyltransferase
pisd: phosphatidylserine decarboxylase
plpp: phosphatidate phosphatase
pmt.: phosphoethanolamine n-methyltransferase
pss: CDP-diacylglycerol:serine phosphatidyltransferase
PtdCho: Phosphatidylcholine
PtdEtn: Phosphatidylethanolamine
PtdGro: Phosphatidylglycerol
PtdIns: Phosphatidylinositol
PtdOH: Phosphatidic acid
PtdSer: Phosphatidylserine
ptdss: phosphatidylserine synthase
sar1: secretion associated, RAS related GTPase 1
TAG: Triacylglycerol
taz: tafazzin
