# Supplementary material for: Transcriptional development of phospholipid and lipoprotein metabolism in different intestinal regions of Atlantic salmon (Salmo salar) fry
Source: BMC Genomics. 2018 Apr 16;19:253. doi: 10.1186/s12864-018-4651-8 (PMC5902856; doi:10.1186/s12864-018-4651-8)
Supplement: Supplementary file 1 — Table S1. Composition and nutritional value of the diet used in current experiment. (DOCX 15 kb) [file 12864_2018_4651_MOESM1_ESM.docx]

**Table S1 Composition and nutritional value of the diet**

| **Feed composition (% of dry weight)** | |
| --- | --- |
| North Atlantic fishmmeal | 40.9 |
| Plant meals | 45.9 |
| Additives | 3.3 |
| North Atlantic fish oil | 9.9 |
| **Nutritional composition (% of diet)** | |
| Protein | 56 |
| Fat | 16 |
| EPA+DHA | 2.2 |
