# Supplementary material for: Transcriptional development of phospholipid and lipoprotein metabolism in different intestinal regions of Atlantic salmon (Salmo salar) fry
Source: BMC Genomics. 2018 Apr 16;19:253. doi: 10.1186/s12864-018-4651-8 (PMC5902856; doi:10.1186/s12864-018-4651-8)
Supplement: Supplementary file 2 — Table S1. List of Atlantic salmon (Ssa) genes involved in phospholipid (PL) de-novo synthesis, lyso-PL synthesis, and lipoprotein (LP) formation pathways. Nomenclature of salmon genes was based on their human (Hsa) and zebrafish (Dre) orthologs. Numbers after underline in Ssa names indicate salmon-specific gene duplicates. NCBI gene ID of salmon and zebrafish is also listed in table. Reference listed the origin of zebrafish genes used for identification of salmon genes. (DOCX 29 kb) [file 12864_2018_4651_MOESM2_ESM.docx]

**Table S1** List of Atlantic salmon (Ssa) genes involved in phospholipid and lipoprotein synthesis pathways.

| **Family** | **Name Hsa** | **Name**  **Dre** | **Name**  **Ssa** | **GeneID Dre** | **GeneID Ssa** | **Chromo-**  **some** | **Reference** | | |
| --- | --- | --- | --- | --- | --- | --- | --- | --- | --- |
| agpat | LPAAT2 | agpat2 | agpat2_1 | 777624 | 106562956 | 11 | kegg database |  |  |
|  | LPAAT2 | agpat2 | agpat2_2 | 777624 | 106568861 | 1 | kegg database | |  |
|  | AGPAT3 | agpat3 | agpat3a_1 | 406734 | 100195109 | 17 | kegg database | |  |
|  | AGPAT3 | agpat3 | agpat3a_2 | 406734 | 106574312 | 16 | kegg database | |  |
|  | AGPAT3 | agpat3 | agpat3b_1 | 406734 | 106582438 | 21 | kegg database | |  |
|  | AGPAT3 | agpat3 | agpat3b_2 | 406734 | 106586274 | 25 | kegg database | |  |
|  | AGPAT4 | agpat4 | agpat4_1 | 406265 | 106589751 | 28 | kegg database | |  |
|  | AGPAT4 | agpat4 | agpat4_2 | 406265 | 100196235 | 1 | kegg database | |  |
|  | AGPAT5 | agpat5 | agpat5_1 | 767778 | 106589814 | 28 | kegg database | |  |
|  | AGPAT5 | agpat5 | agpat5_2 | 767778 | 106612554 | 1 | kegg database | |  |
| apoa1 | APOA1 | apoa1 | apoa1_1 | 30355 | 100136573 | 20 | Otis JP, *et al.* (2015) | |  |
|  | APOA1 | apoa1 | apoa1_2 | 30355 | 100196111 | 9 | Otis JP, *et al.* (2015) | |  |
| apoa4 | APOA4 | apoa4a | apoa4a_1 | 550608 | 106586541 | 25 | Otis JP, *et al.* (2015) | |  |
|  | APOA4 | apoa4a | apoa4b_2 | 550608 | 106569632 | 1 | Otis JP, *et al.* (2015) | |  |
|  | APOA4 | apoa4b.2 | apoa4b_1 | 570354 | 106577499 | 2 | Otis JP, *et al.* (2015) | |  |
|  | APOA4 | apoa4b.2 | apoa4b_2 | 570354 | 106605692 | 5 | Otis JP, *et al.* (2015) | |  |
|  | APOA4 | apoa4b.2 | apoa4c_1 | 570354 | 106577505 | 2 | Otis JP, *et al.* (2015) | |  |
|  | APOA4 | apoa4b.2 | apoa4c_2 | 570354 | 106605691 | 5 | Otis JP, *et al.* (2015) | |  |
| apob | APOB | apoba | apoba | 566465 | 106611125 | 9 | Otis JP, *et al.* (2015) | |  |
|  | APOB | apoba | apobb | 566465 | 106571178 | 15 | Otis JP, *et al.* (2015) | |  |
|  | APOB | apobb | apobc | 321166 | 100136579 | 15 | Otis JP, *et al.* (2015) | |  |
| cd36 | CD36 | cd36 | cd36_1 | 436636 | 106576427 | 17 | kegg database | |  |
|  | CD36 | cd36 | cd36_2 | 436636 | 106609495 | 7 | kegg database | |  |
| cdipt | CDIPT | cdipt | cdipt | 404620 | 106600754 | 3 | kegg database | |  |
| cds | CDS1 | cds1 | cds1a | 100002015 | 106570914 | 15 | kegg database | |  |
|  | CDS1 | cds1 | cds1b | 100002015 | 106585948 | 24 | kegg database | |  |
|  | CDS2 | cds2 | cds2_1 | 394161 | 106580500 | 20 | kegg database | |  |
|  | CDS2 | cds2 | cds2_2 | 394161 | 106585108 | 24 | kegg database | |  |
| cept | CEPT1 | cept1b | cept1a_1 | 570720 | 100194840 | 12 | kegg database | |  |
|  | CEPT1 | cept1b | cept1a_2 | 570720 | 106583483 | 22 | kegg database | |  |
|  | CEPT1 | cept1b | cept1b_1 | 570720 | 106566938 | 13 | kegg database | |  |
|  | CEPT1 | cept1b | cept1b_2 | 570720 | 106572169 | 15 | kegg database | |  |
| chk | CHKA | chka | chka_1 | 558499 | 106573758 | 16 | kegg database | |  |
|  | CHKA | chka | chka_2 | 558499 | 106560813 | 10 | kegg database | | |
|  | CHKB | chkb | chkb | 563589 | 106561237 | 10 | kegg database | | |
| chpt | CHPT1 | chpt1 | chpt1_1 | 322605 | 106609452 | 7 | kegg database | | |
|  | CHPT1 | chpt1 | chpt1_2 | 322605 | 106576273 | 17 | kegg database | | |
|  | CRLS1 | crls1 | crls1 | 405867 | 100380602 | 1 | kegg database | | |
| ept | EPT1 | si:ch211-197l9.4 | ept1_a | 557632 | 106594620 | Unknown | kegg database | | |
|  | EPT1 | si:ch211-197l9.4 | ept1_b | 557632 | 106571011 | 15 | kegg database | | |
| etnk | ETNK1 | etnk1 | etnk1_1 | 565971 | 106576151 | 17 | kegg database | | |
|  | ETNK1 | etnk1 | etnk1_2 | 565971 | 106609326 | 7 | kegg database | | |
|  | ETNK2 | etnk2 | etnk2_1 | 555330 | 106583121 | 22 | kegg database | | |
|  | ETNK2 | etnk2 | etnk2_2 | 555330 | 106565623 | 12 | kegg database | | |
| fabp | fabp1 | fabp1 | fabp1 | 791610 | 106563252 | 11 | Neeli I, *et al*. (2007) | | |
| gpat | GPAT1 | gpatm | gpat1 | 100332911 | 106579146 | 19 | kegg database | | |
|  | GPAT2 | gpat2 | gpat2 | 558437 | 106580447 | 20 | kegg database | | |
|  | GPAT3 | gpat3a | gpat3a_1 | 436958 | 100196460 | 13 | kegg database | | |
|  | GPAT3 | gpat3a | gpat3a_2 | 436958 | 106563657 | 1 | kegg database | | |
|  | GPAT3 | gpat3b | gpat3b_1 | 567414 | 106580584 | 20 | kegg database | | |
|  | GPAT3 | gpat3b | gpat3b_2 | 567414 | 106585165 | 24 | kegg database | | |
|  | GPAT4 | gpat4 | gpat4a | 678522 | 106568233 | 13 | kegg database | | |
|  | GPAT4 | gpat4 | gpat4b_1 | 678522 | 106585610 | 24 | kegg database | | |
|  | GPAT4 | gpat4 | gpat4b_2 | 678522 | 100195147 | 20 | kegg database | | |
| lclat | LCLAT1 | lycat | lclat1 | 406554 | 106601632 | 1 | kegg database | | |
| lpcat | LPCAT1 | lpcat1 | lpcat1a | 555969 | 106570327 | 14 | kegg database | | |
|  | LPCAT1 | lpcat1 | lpcat1b_1 | 555969 | 106592264 | Unknown | kegg database | | |
|  | LPCAT1 | lpcat1 | lpcat1b_2 | 555969 | 106597556 | Unknown | kegg database | | |
|  | LPCAT2 | lpcat2 | lpcat2_1 | 553683 | 100380789 | 11 | kegg database | | |
|  | LPCAT2 | lpcat2 | lpcat2_2 | 553683 | 106562418 | 11 | kegg database | | |
|  | LPCAT3 | lpcat3 | lpcat3_1 | 555566 | 106583352 | 2 | kegg database | | |
|  | LPCAT3 | lpcat3 | lpcat3_2 | 555566 | 106605098 | 5 | kegg database | | |
|  | LPEAT2 | lpcat4 | lpcat4_1 | 327566 | 100196731 | 7 | kegg database | | |
|  | LPEAT2 | lpcat4 | lpcat4_2 | 327566 | 106577939 | 18 | kegg database | | |
| lpgat | LPGAT1 | lpgat1 | lpgat1_1 | 561832 | 106571200 | 15 | kegg database | | |
|  | LPGAT1 | lpgat1 | lpgat1_2 | 561832 | 106607581 | 6 | kegg database | | |
|  | LPIAT1 | mboat7 | lpiat1 | 393509 | 106577207 | 2 | kegg database | | |
| lpin | LPIN1 | lpin1 | lpin1_1 | 799099 | 106570052 | 1 | kegg database | | |
|  | LPIN1 | lpin1 | lpin1_2 | 799099 | 106610734 | 9 | kegg database | | |
|  | LPIN2 | lipin2 | lpin2_1 | 558422 | 106578699 | 19 | kegg database | | |
|  | LPIN2 | lipin2 | lpin2_2 | 558422 | 106590143 | 29 | kegg database | | |
|  | LPIN3 | zgc:123305 | lpin3_1 | 641489 | 106583597 | 22 | kegg database | | |
|  | LPIN3 | zgc:123305 | lpin3_2 | 641489 | 106565215 | 12 | kegg database | | |
| mboat | LPEAT2 | mboat2a | mboat2a_1 | 570695 | 106566507 | 1 | kegg database | | |
|  | LPEAT2 | mboat2a | mboat2a_2 | 570695 | 106610702 | 9 | kegg database | | |
|  | LPEAT2 | mboat2b | mboat2b_1 | 692278 | 106571255 | 15 | kegg database | | |
|  | LPEAT2 | mboat2b | mboat2b_2 | 692278 | 106607658 | 6 | kegg database | | |
| mtp | MTP | mtp | mtp_1 | 406207 | 106602715 | 4 | Schlegel A and Stainier DY (2006) | | |
|  | MTP | mtp | mtp_2 | 406207 | 106609996 | 8 | Schlegel A and Stainier DY (2006) | | |
| pcyt1 | PCYT1A | pcyt1a | pcyt1aa | 550327 | 106612410 | 9 | kegg database | | |
|  | PCYT1A | pcyt1a | pcyt1ab_1 | 553770 | 106568933 | 14 | kegg database | | |
|  | PCYT1A | pcyt1a | pcyt1ab_2 | 553770 | 106599064 | 3 | kegg database | | |
|  | PCYT1B | pcyt1ba | pcyt1ba_1 | 100001552 | 106578451 | 19 | kegg database | | |
|  | PCYT1B | pcyt1ba | pcyt1ba_2 | 100001552 | 106590595 | 29 | kegg database | | |
|  | PCYT1B | pcyt1bb | pcyt1bb_1 | 555725 | 106603074 | 4 | kegg database | | |
|  | PCYT1B | pcyt1bb | pcyt1bb_2 | 555725 | 106563634 | 11 | kegg database | | |
| pcyt2 | PCYT2 | pcyt2 | pcyt2a | 450016 | 100380284 | 2 | kegg database | | |
|  | PCYT2 | pcyt2 | pcyt2b | 450016 | 106606425 | 6 | kegg database | | |
|  | PCYT2 | pcyt2 | pcyt2c_1 | 450016 | 106601930 | 6 | kegg database | | |
|  | PCYT2 | pcyt2 | pcyt2c_2 | 450016 | 106606443 | 3 | kegg database | | |
| pemt | PEMT | pemt | pemt | 393127 | 100196273 | 19 | kegg database | | |
| pgs | PGS1 | pgs1 | pgs1_1 | 100534896 | 100380610 | 1 | kegg database | | |
|  | PGS1 | pgs1 | pgs1_2 | 100534896 | 106589835 | 28 | kegg database | | |
| pisd | PISD | pisd | pisd_1 | 553433 | 106563023 | 11 | kegg database | | |
|  | PISD | pisd | pisd_2 | 553433 | 100380321 | 1 | kegg database | | |
| plpp | PLPP1 | LOC794598 | plpp1a_1 | 794598 | 106580570 | 20 | kegg database | | |
|  | PLPP1 | LOC794598 | plpp1a_2 | 794598 | 106585174 | 24 | kegg database | | |
|  | PLPP1 | si:ch73-96j23.1 | plpp1b_1 | 569053 | 106561845 | 1 | kegg database | | |
|  | PLPP1 | si:ch73-96j23.1 | plpp1b_2 | 569053 | 106568407 | 13 | kegg database | | |
|  | PLPP2 | LOC563806 | plpp2a_1 | 563806 | 106574497 | 16 | kegg database | | |
|  | PLPP2 | LOC563806 | plpp2a_2 | 563806 | 106575392 | 17 | kegg database | | |
|  | PLPP2 | plpp2b | plpp2b_1 | 335485 | 106584410 | 23 | kegg database | | |
|  | PLPP2 | plpp2b | plpp2b_2 | 335485 | 100195687 | 10 | kegg database | | |
|  | PLPP3 | plpp3 | plpp3a_1 | 557680 | 106584209 | 23 | kegg database | | |
|  | PLPP3 | plpp3 | plpp3a_2 | 557680 | 106560230 | 10 | kegg database | | |
|  | PLPP3 | plpp3 | plpp3b_1 | 557680 | 106569302 | 14 | kegg database | | |
|  | PLPP3 | plpp3 | plpp3b_2 | 557680 | 106600418 | 3 | kegg database | | |
|  | PLPP3 | ppap2d | plpp3c | 559124 | 106592432 | 2 | kegg database | | |
|  | PLPP3 | ppap2d | plpp3d | 559124 | 106606828 | 6 | kegg database | | |
| pmt | NA | pmt | pmt_1 | 767699 | 106594428 | Unknown | kegg database | | |
|  | NA | pmt | pmt_2 | 767699 | 100380594 | 5 | kegg database | | |
| pss | NA | PSS | pss | 559970 | 100286735 | 15 | Carmona AG, *et al.* (2015) | | |
| ptdss1 | PTDSS1 | ptdss1a | ptdss1a | 393931 | 106595791 | Unknown | kegg database | | |
|  | PTDSS1 | ptdss1a | ptdss1b | 393931 | 106590970 | Unknown | kegg database | | |
|  | PTDSS1 | ptdss1b | ptdss1c_1 | 559442 | 106570478 | 14 | kegg database | | |
|  | PTDSS1 | ptdss1b | ptdss1c_2 | 559442 | 106588913 | 27 | kegg database | | |
| ptdss2 | PTDSS2 | ptdss2 | ptdss2 | 100004136 | 106560699 | 10 | kegg database | | |
| sar1 | SAR1A | sar1a | sar1a_1 | 550581 | 106608453 | 1 | Levic DS, *et al.* (2015) | | |
|  | SAR1A | sar1a | sar1a_2 | 550581 | 106577216 | 18 | Levic DS, *et al.* (2015) | | |
|  | SAR1A | sar1a | sar1ba_1 | 554177 | 100196767 | 4 | Levic DS, *et al.* (2015) | | |
|  | SAR1B | sar1b | sar1ba_2 | 554177 | 106567531 | 13 | Levic DS, *et al.* (2015) | | |
|  | SAR1A | sar1a | sar1bb_1 | 554177 | 106560851 | 1 | Levic DS, *et al.* (2015) | | |
|  | SAR1B | sar1b | sar1bb_2 | 554177 | 106577347 | 18 | Levic DS, *et al.* (2015) | | |
| taz | TAZ | taz | taz_1 | 321965 | 100196028 | 15 | kegg database | | |
|  | TAZ | taz | taz_2 | 321965 | 106572982 | 15 | kegg database | | |

The PL and LP synthesis pathways are phospholipid (PL) *de-novo* synthesis, lyso-PL synthesis and lipoprotein (LP) formation pathways. Nomenclature of salmon genes was based on their human (Hsa) and zebrafish (Dre) paralogs. Numbers after underline in Ssa names indicate salmon-specific gene duplicates. NCBI gene ID of salmon and zebrafish is also listed in table. Reference listed the origin of zebrafish genes used for identification of salmon genes.
