# Supplementary material for: Transcriptional development of phospholipid and lipoprotein metabolism in different intestinal regions of Atlantic salmon (Salmo salar) fry
Source: BMC Genomics. 2018 Apr 16;19:253. doi: 10.1186/s12864-018-4651-8 (PMC5902856; doi:10.1186/s12864-018-4651-8)
Supplement: Supplementary file 3 — Table S1. Summary of mapping statistics of all 45 samples used for RNA sequencing (2 fish each replicate × 3 replicates × 3 tissue in 0.16 g fish, 1 fish each replicate × 6 replicates × 3 tissue in 2.5 and 10 g fish). (DOCX 16 kb) [file 12864_2018_4651_MOESM3_ESM.docx]

**Table S1** Summary of mapping statistics of all 45 samples used for RNA sequencing (2 fish each replicate x 3 replicates x 3 tissue in 0.16g fish, 1 fish each replicate x 6 replicates x 3 tissue in 2.5g and 10g fish).

| **Mapping Statistics** | **Mean±sd** |
| --- | --- |
| Total number of libraries | 45 |
| Average input read length | 100.8±3.1 |
| Number of input reads | 225867563±5672675 |
| Number of mapped reads | 19031474±4676927 |
| Number of reads mapped to too many loci | 208432±73356 |
| Percentage of mapped reads | 84.5%±4% |
| Percentage of reads mapped to multiple loci | 11.3%±4% |
| Percentage of reads mapped to too many loci | 0.9%±0% |
| Percentage of reads unmapped: too short | 1.4%±0% |
| Percentage of reads unmapped: other | 1.8%±2% |
| Total | 100 % |

Average value of all samples was given in table.
