# Supplementary material for: Transcriptional development of phospholipid and lipoprotein metabolism in different intestinal regions of Atlantic salmon (Salmo salar) fry
Source: BMC Genomics. 2018 Apr 16;19:253. doi: 10.1186/s12864-018-4651-8 (PMC5902856; doi:10.1186/s12864-018-4651-8)
Supplement: Supplementary file 6 — Table S1. Transcript per million (TPM) of all gene duplicates in phospholipid and lipoprotein synthesis pathways in stomach, pyloric caeca, and Hindgut of 0.16, 2.5 and 10 g salmon. (DOCX 32 kb) [file 12864_2018_4651_MOESM6_ESM.docx]

**Table S1** Transcript per million (TPM) of all gene duplicates in phospholipid and lipoprotein synthesis pathways in stomach, pyloric caeca and Hindgut of 0.16g, 2.5g and 10g salmon.

|  | **Stomach** | | | **Pyloric caeca** | | | **Hindgut** | | |
| --- | --- | --- | --- | --- | --- | --- | --- | --- | --- |
| **Name** | **0.16g** | **2.5g** | **10g** | **0.16g** | **2.5g** | **10g** | **0.16g** | **2.5g** | **10g** |
| agpat2_1 | 1.5 | 2.6 | 1.7 | 1.6 | 0.9 | 1.0 | 0.8 | 1.2 | 1.2 |
| agpat2_2 | 3.1 | 4.1 | 3.3 | 12.8 | 16.6 | 18.9 | 5.9 | 16.8 | 18.6 |
| agpat3a_1 | 42.3 | 32.5 | 29.9 | 156 | 172 | 240.7 | 145 | 105 | 103 |
| agpat3a_2 | 19.8 | 16.3 | 13.4 | 31.7 | 23.5 | 32.4 | 28.0 | 17.5 | 16.7 |
| agpat3b_1 | 6.7 | 5.3 | 4.1 | 4.8 | 2.8 | 2.3 | 2.9 | 2.8 | 3.1 |
| agpat3b_2 | 5.7 | 5.0 | 4.7 | 5.0 | 4.1 | 2.9 | 3.9 | 3.6 | 3.7 |
| agpat4_1 | 12.6 | 24.6 | 23.9 | 2.5 | 1.3 | 0.9 | 2.4 | 2.7 | 2.1 |
| agpat4_2 | 18.3 | 34.2 | 22.8 | 95.2 | 164 | 196 | 55.0 | 64.1 | 65.4 |
| agpat5_1 | 15.1 | 21.5 | 22.1 | 7.1 | 7.8 | 7.2 | 3.7 | 4.5 | 4.3 |
| agpat5_2 | 1.6 | 2.2 | 1.6 | 2.0 | 2.1 | 1.9 | 1.3 | 1.5 | 1.6 |
| apoa1_1 | 7.7 | 30.8 | 40.8 | 2065 | 14611 | 26968 | 265 | 1313 | 1554 |
| apoa1_2 | 16.2 | 40.9 | 19.8 | 1071 | 2750 | 8217 | 826 | 255 | 591 |
| apoa4a_1 | 0.8 | 11.3 | 3.0 | 53.8 | 1118 | 4157 | 162.2 | 178.6 | 338.0 |
| apoa4b_1 | 1.8 | 3.7 | 3.4 | 262 | 638 | 1516 | 77.8 | 123 | 216 |
| apoa4b_2 | 11.7 | 20.3 | 17.3 | 390 | 2122 | 5046 | 183 | 324 | 418 |
| apoa4b_2 | 0.2 | 3.0 | 1.6 | 60.5 | 169 | 811 | 62.8 | 46.6 | 88.7 |
| apoa4c_1 | 1.9 | 13.7 | 14.9 | 1878 | 4494 | 7988 | 281 | 887 | 1169 |
| apoa4c_2 | 0.1 | 0.9 | 1.1 | 16.0 | 332 | 798 | 56.3 | 64.3 | 93.2 |
| Apoba | 0.2 | 0.3 | 0.1 | 0.5 | 0.1 | 0.3 | 10.4 | 0.1 | 0.2 |
| Apobb | 3.6 | 12.0 | 12.1 | 877 | 1860 | 2677 | 92.5 | 426 | 497 |
| Apobc | 1.6 | 2.6 | 0.8 | 23.7 | 2.1 | 3.9 | 114.3 | 0.9 | 1.6 |
| cd36_1 | 1.3 | 1.7 | 2.4 | 532 | 785 | 1000 | 19.0 | 133 | 99.6 |
| cd36_2 | 0.1 | 0.2 | 0.2 | 29.8 | 177 | 117 | 0.8 | 49.6 | 36.2 |
| Cdipt | 21.8 | 26.3 | 26.4 | 21.4 | 27.0 | 28.9 | 23.6 | 26.0 | 25.6 |
| cds1a | 0.4 | 0.2 | 0.2 | 2.0 | 2.5 | 1.3 | 0.6 | 0.7 | 0.5 |
| cds1b | 4.7 | 4.5 | 3.3 | 16.1 | 19.0 | 20.3 | 14.5 | 16.9 | 15.3 |
| cds2_1 | 3.3 | 3.9 | 4.2 | 2.5 | 2.0 | 1.6 | 2.8 | 2.4 | 2.1 |
| cds2_2 | 5.1 | 3.9 | 4.5 | 4.5 | 2.5 | 2.1 | 4.4 | 3.1 | 2.6 |
| cept1a_1 | 9.4 | 9.7 | 9.6 | 11.5 | 19.4 | 19.5 | 10.3 | 17.9 | 20.5 |
| cept1a_2 | 7.9 | 8.3 | 8.1 | 5.8 | 8.6 | 7.2 | 10.2 | 9.6 | 10.9 |
| cept1b_1 | 8.9 | 7.9 | 8.3 | 9.5 | 10.9 | 12.0 | 8.4 | 9.3 | 8.7 |
| cept1b_2 | 15.9 | 14.6 | 12.5 | 6.7 | 7.1 | 5.9 | 5.3 | 6.0 | 6.2 |
| chka_1 | 2.1 | 9.3 | 9.1 | 11.1 | 10.2 | 20.7 | 4.7 | 1.6 | 1.0 |
| chka_2 | 5.3 | 4.7 | 5.0 | 16.6 | 7.9 | 15.4 | 9.2 | 4.7 | 6.1 |
| Chkb | 8.3 | 7.7 | 8.2 | 17.9 | 54.4 | 89.9 | 13.8 | 21.3 | 21.1 |
| chpt1_1 | 10.1 | 13.1 | 11.4 | 64.2 | 131 | 185 | 29.2 | 52.5 | 56.6 |
| chpt1_2 | 5.2 | 3.4 | 3.8 | 58.5 | 80.0 | 106 | 33.4 | 40.1 | 37.5 |
| crls1 | 14.4 | 17.7 | 17.9 | 19.2 | 20.6 | 26.1 | 7.6 | 12.0 | 11.7 |
| ept1_1 | 2.9 | 6.9 | 3.6 | 5.8 | 12.3 | 15.5 | 6.8 | 12.5 | 13.0 |
| ept1_2 | 2.7 | 5.9 | 4.2 | 5.4 | 9.7 | 12.8 | 5.5 | 9.1 | 11.0 |
| etnk1_1 | 78.7 | 94.3 | 101 | 29.2 | 30.0 | 23.4 | 46.4 | 34.6 | 36.5 |
| etnk1_2 | 26.1 | 20.5 | 22.2 | 21.5 | 18.4 | 15.1 | 54.3 | 21.7 | 20.6 |
| etnk2_1 | 0.6 | 2.2 | 1.2 | 1.4 | 2.1 | 2.4 | 3.5 | 3.8 | 4.1 |
| etnk2_2 | 9.1 | 15.2 | 11.2 | 14.1 | 58.7 | 109.4 | 4.6 | 30.0 | 33.0 |
| fabp1 | 2.6 | 3.3 | 11.1 | 1998 | 2548 | 7203 | 1222 | 751 | 842 |
| gpat1 | 7.6 | 11.1 | 8.9 | 31.4 | 40.5 | 50.3 | 9.0 | 17.8 | 18.6 |
| gpat2 | 5.5 | 5.6 | 5.1 | 6.7 | 5.2 | 4.7 | 7.6 | 4.1 | 3.9 |
| gpat3a_1 | 11.4 | 20.5 | 13.5 | 7.0 | 5.9 | 4.6 | 7.1 | 7.0 | 6.6 |
| gpat3a_2 | 18.1 | 14.4 | 13.5 | 8.9 | 7.1 | 6.3 | 8.0 | 5.9 | 6.1 |
| gpat3b_1 | 5.2 | 18.2 | 17.4 | 85.8 | 100 | 127 | 45.6 | 62.1 | 54.8 |
| gpat3b_2 | 20.9 | 29.1 | 28.4 | 55.4 | 77.9 | 108 | 63.0 | 53.9 | 58.1 |
| gpat4a | 0.2 | 0.1 | 0.2 | 0.1 | 0.0 | 0.0 | 0.1 | 0.0 | 0.0 |
| gpat4b_1 | 4.7 | 3.1 | 2.7 | 3.0 | 2.3 | 1.6 | 3.0 | 2.2 | 2.0 |
| gpat4b_2 | 1.1 | 0.9 | 0.8 | 1.2 | 0.8 | 0.6 | 1.4 | 1.0 | 0.9 |
| lclat1 | 10.8 | 10.9 | 10.7 | 10.9 | 9.8 | 10.8 | 10.9 | 10.6 | 10.5 |
| lpcat1a | 7.2 | 5.2 | 7.4 | 8.1 | 9.0 | 7.3 | 14.5 | 9.7 | 10.7 |
| lpcat1b_1 | 0.2 | 0.2 | 0.3 | 0.1 | 0.3 | 0.3 | 0.1 | 0.4 | 0.6 |
| lpcat1b_2 | 0.0 | 0.0 | 0.0 | 0.0 | 0.0 | 0.1 | 0.0 | 0.1 | 0.1 |
| lpcat2_1 | 12.8 | 10.2 | 11.8 | 11.3 | 11.3 | 8.8 | 10.3 | 8.4 | 7.3 |
| lpcat2_2 | 9.5 | 7.1 | 8.4 | 6.9 | 7.7 | 6.0 | 5.7 | 6.7 | 6.4 |
| lpcat3_1 | 6.7 | 12.5 | 6.7 | 71.2 | 131 | 197 | 11.8 | 25.5 | 21.2 |
| lpcat3_2 | 3.1 | 4.5 | 3.2 | 8.8 | 17.3 | 28.4 | 5.2 | 7.4 | 7.6 |
| lpcat4_1 | 20.8 | 25.6 | 26.6 | 24.8 | 27.9 | 31.5 | 22.8 | 23.0 | 26.4 |
| lpcat4_2 | 0.9 | 0.0 | 0.0 | 0.0 | 0.0 | 0.0 | 0.4 | 0.1 | 0.0 |
| lpgat1_1 | 1.6 | 2.3 | 1.8 | 4.8 | 12.6 | 18.8 | 4.7 | 8.4 | 9.5 |
| lpgat1_2 | 1.0 | 1.1 | 0.9 | 2.1 | 3.2 | 4.2 | 1.8 | 3.1 | 3.2 |
| lpiat1 | 16.2 | 20.5 | 18.4 | 14.1 | 24.0 | 26.6 | 16.2 | 23.0 | 22.7 |
| lpin1_1 | 0.5 | 0.5 | 0.3 | 0.5 | 0.3 | 0.3 | 0.6 | 0.3 | 0.3 |
| lpin1_2 | 6.0 | 3.6 | 2.5 | 12.4 | 5.3 | 2.7 | 9.1 | 5.4 | 4.4 |
| lpin2_1 | 0.6 | 0.9 | 0.9 | 2.9 | 5.6 | 6.4 | 4.3 | 8.8 | 8.7 |
| lpin2_2 | 23.8 | 12.8 | 14.7 | 20.1 | 19.7 | 21.4 | 35.1 | 33.8 | 39.3 |
| lpin3_1 | 2.1 | 2.8 | 1.4 | 3.1 | 2.7 | 2.8 | 3.6 | 2.8 | 2.3 |
| lpin3_2 | 8.9 | 11.6 | 8.7 | 12.6 | 28.9 | 35.1 | 15.2 | 20.1 | 19.0 |
| mboat2a_1 | 23.6 | 24.4 | 23.0 | 8.1 | 3.7 | 3.7 | 14.3 | 9.9 | 10.9 |
| mboat2a_2 | 11.9 | 17.8 | 21.5 | 8.7 | 5.0 | 9.8 | 18.2 | 21.3 | 28.3 |
| mboat2b_1 | 4.4 | 4.4 | 5.5 | 1.9 | 1.9 | 1.9 | 2.2 | 2.6 | 2.3 |
| mboat2b_2 | 9.6 | 5.4 | 9.9 | 6.7 | 2.5 | 2.1 | 6.8 | 3.1 | 2.8 |
| mtp_1 | 7.2 | 7.1 | 6.5 | 94.6 | 177 | 242 | 34.6 | 68.6 | 80.8 |
| mtp_2 | 0.1 | 0.1 | 0.2 | 65.6 | 105 | 176 | 2.6 | 7.9 | 8.1 |
| pcyt1aa | 6.0 | 8.6 | 7.4 | 6.3 | 6.8 | 5.7 | 8.9 | 9.3 | 9.0 |
| pcyt1ab_1 | 7.4 | 14.5 | 11.5 | 2.8 | 6.5 | 9.1 | 2.7 | 5.9 | 7.1 |
| pcyt1ab_2 | 13.2 | 10.8 | 11.6 | 15.9 | 15.7 | 16.2 | 16.2 | 15.5 | 16.5 |
| pcyt1ba_1 | 2.3 | 3.8 | 4.1 | 0.8 | 1.3 | 1.1 | 0.5 | 1.1 | 1.1 |
| pcyt1ba_2 | 0.5 | 0.5 | 0.4 | 0.3 | 0.4 | 0.2 | 0.6 | 0.5 | 0.4 |
| pcyt1bb_1 | 0.4 | 0.6 | 0.8 | 56.9 | 82.4 | 128 | 15.9 | 23.3 | 29.3 |
| pcyt1bb_2 | 0.0 | 0.0 | 0.1 | 16.5 | 22.7 | 44.3 | 4.9 | 6.2 | 8.3 |
| pcyt2a | 20.2 | 25.3 | 15.2 | 37.2 | 43.0 | 64.1 | 41.8 | 37.5 | 43.9 |
| pcyt2b | 0.5 | 0.7 | 1.1 | 0.9 | 0.9 | 3.5 | 3.0 | 7.2 | 7.6 |
| pcyt2c_1 | 0.4 | 0.7 | 1.1 | 0.3 | 1.3 | 5.6 | 0.8 | 2.9 | 4.8 |
| pcyt2c_2 | 0.3 | 0.9 | 1.0 | 0.4 | 2.2 | 7.1 | 0.3 | 2.5 | 4.3 |
| Pemt | 17.4 | 19.2 | 19.0 | 26.7 | 30.1 | 38.4 | 20.6 | 20.5 | 19.3 |
| pgs1_1 | 10.8 | 7.2 | 7.2 | 18.7 | 12.0 | 15.0 | 7.0 | 6.9 | 6.2 |
| pgs1_2 | 4.5 | 3.6 | 3.2 | 4.4 | 4.0 | 3.8 | 3.2 | 3.5 | 3.2 |
| pisd_1 | 8.4 | 8.3 | 9.0 | 10.1 | 9.0 | 11.2 | 7.8 | 8.5 | 9.3 |
| pisd_2 | 10.1 | 7.7 | 6.0 | 3.4 | 2.5 | 2.9 | 6.2 | 3.2 | 3.5 |
| plpp1a_1 | 0.5 | 1.5 | 1.5 | 0.3 | 0.7 | 0.2 | 0.2 | 0.7 | 0.5 |
| plpp1a_2 | 1.0 | 5.9 | 4.3 | 3.1 | 30.9 | 22.1 | 0.9 | 14.6 | 13.1 |
| plpp1b_1 | 26.7 | 30.5 | 31.4 | 38.5 | 37.8 | 41.4 | 24.5 | 24.8 | 24.3 |
| plpp1b_2 | 20.4 | 26.2 | 26.8 | 26.5 | 33.5 | 37.0 | 29.0 | 34.2 | 34.5 |
| plpp2a_1 | 3.7 | 5.6 | 3.1 | 2.3 | 1.5 | 1.6 | 2.1 | 1.9 | 1.5 |
| plpp2a_2 | 8.9 | 10.6 | 7.2 | 6.6 | 8.8 | 6.3 | 9.5 | 13.8 | 13.8 |
| plpp2b_1 | 6.1 | 9.8 | 8.3 | 6.9 | 8.1 | 8.7 | 9.2 | 12.1 | 11.6 |
| plpp2b_2 | 9.5 | 7.3 | 10.7 | 9.3 | 12.1 | 16.2 | 11.3 | 8.9 | 11.1 |
| plpp3a_1 | 3.1 | 3.4 | 3.2 | 2.9 | 3.5 | 2.5 | 2.3 | 2.9 | 3.0 |
| plpp3a_2 | 11.8 | 15.6 | 14.5 | 21.3 | 18.6 | 15.5 | 30.0 | 16.4 | 17.3 |
| plpp3b_1 | 5.8 | 4.2 | 5.1 | 4.4 | 3.6 | 3.7 | 3.1 | 3.5 | 3.4 |
| plpp3b_2 | 12.5 | 10.9 | 16.6 | 6.8 | 4.3 | 3.6 | 5.1 | 5.1 | 4.5 |
| plpp3c | 0.4 | 0.6 | 0.6 | 1.5 | 1.0 | 1.2 | 0.7 | 0.9 | 0.7 |
| plpp3d | 4.0 | 7.6 | 7.7 | 19.1 | 18.1 | 19.3 | 11.1 | 13.1 | 11.9 |
| pmt_1 | 0.5 | 7.6 | 1.4 | 0.3 | 2.4 | 0.9 | 0.5 | 0.2 | 0.1 |
| pmt_2 | 4.6 | 49.6 | 34.3 | 8.2 | 8.2 | 6.8 | 20.8 | 0.5 | 0.5 |
| Pss | 0.7 | 0.2 | 0.3 | 0.5 | 0.1 | 0.1 | 1.2 | 0.3 | 0.4 |
| ptdss1a | 0.7 | 0.6 | 0.6 | 0.5 | 0.6 | 0.4 | 1.0 | 1.0 | 0.7 |
| ptdss1b | 5.8 | 9.4 | 5.7 | 5.6 | 7.0 | 6.4 | 12.0 | 10.0 | 9.4 |
| ptdss1c_1 | 0.5 | 1.0 | 0.7 | 1.3 | 0.9 | 1.0 | 1.5 | 1.6 | 1.4 |
| ptdss1c_2 | 1.6 | 0.6 | 0.6 | 1.4 | 0.8 | 0.8 | 0.9 | 0.8 | 0.5 |
| ptdss2 | 2.4 | 3.6 | 2.4 | 4.8 | 3.5 | 4.2 | 2.6 | 2.9 | 3.3 |
| sar1a_1 | 1.1 | 0.6 | 0.3 | 0.2 | 0.2 | 0.3 | 0.6 | 0.2 | 0.2 |
| sar1a_2 | 1.8 | 1.9 | 2.0 | 4.3 | 7.8 | 10.5 | 2.3 | 4.3 | 4.1 |
| sar1ba_1 | 57.6 | 96.4 | 80.2 | 91.7 | 235 | 330 | 59.7 | 112 | 112 |
| sar1ba_2 | 50.1 | 79.1 | 64.1 | 89.5 | 179 | 237 | 70.7 | 130 | 129 |
| sar1bb_1 | 3.2 | 4.4 | 4.1 | 5.7 | 9.8 | 13.3 | 6.6 | 8.0 | 9.4 |
| sar1bb_2 | 2.9 | 4.2 | 3.3 | 5.0 | 9.0 | 13.9 | 7.1 | 9.4 | 10.8 |
| taz_1 | 15.2 | 14.1 | 10.7 | 18.5 | 15.1 | 17.5 | 15.6 | 13.9 | 11.6 |
| taz_2 | 28.5 | 20.4 | 19.0 | 33.2 | 25.1 | 25.5 | 28.9 | 17.9 | 17.2 |
