# Supplementary material for: Transcriptional development of phospholipid and lipoprotein metabolism in different intestinal regions of Atlantic salmon (Salmo salar) fry
Source: BMC Genomics. 2018 Apr 16;19:253. doi: 10.1186/s12864-018-4651-8 (PMC5902856; doi:10.1186/s12864-018-4651-8)
Supplement: Supplementary file 7 — Table S1. Log2 fold change (LogFC) and adjusted p value (q) of all gene duplicates in phospholipid and lipoprotein synthesis pathways in stomach, pyloric caeca and hindgut of 2.5 and 10 g salmon both compared to 0.16 g. (DOCX 34 kb) [file 12864_2018_4651_MOESM7_ESM.docx]

**Table S1** Log2 fold change (LogFC) and adjusted *p* value (*q*) of all gene duplicates in phospholipid and lipoprotein synthesis pathways in stomach, pyloric caeca and hindgut of 2.5g and 10g salmon both compared to 0.16g.

|  | **Stomach** | | | | **Pyloric caeca** | | | | **Hindgut** | | | |
| --- | --- | --- | --- | --- | --- | --- | --- | --- | --- | --- | --- | --- |
|  | **2.5g** | | **10g** | | **2.5g** | | **10g** | | **2.5g** | | **10g** | |
| **Name.Ssa** | **logFC** | ***q*** | **logFC** | ***q*** | **logFC** | ***q*** | **logFC** | ***q*** | **logFC** | ***q*** | **logFC** | ***q*** |
| agpat2_1 | 0.96 | 0.22 | 0.42 | 0.74 | -0.64 | 0.24 | -0.51 | 0.30 | 0.63 | 0.13 | 0.63 | 0.13 |
| agpat2_2 | 0.33 | 0.71 | 0.08 | 0.95 | 0.66 | 0.43 | 0.77 | 0.28 | 1.62 | <0.01 | 1.73 | <0.01 |
| agpat3a_1 | -0.24 | 0.68 | -0.34 | 0.62 | 0.00 | 1.00 | 0.46 | 0.09 | -0.50 | 0.01 | -0.53 | <0.01 |
| agpat3a_2 | -0.26 | 0.45 | -0.54 | 0.09 | -0.66 | <0.01 | -0.25 | 0.36 | -0.76 | <0.01 | -0.84 | <0.01 |
| agpat3b_1 | -0.26 | 0.61 | -0.64 | 0.16 | -1.03 | <0.01 | -1.25 | <0.01 | 0.06 | 0.87 | 0.16 | 0.60 |
| agpat3b_2 | -0.11 | 0.81 | -0.19 | 0.70 | -0.34 | 0.38 | -0.83 | 0.01 | -0.09 | 0.79 | -0.04 | 0.91 |
| agpat4_1 | 0.78 | 0.02 | 0.75 | 0.03 | -0.48 | 0.54 | -1.14 | 0.06 | 0.25 | 0.50 | -0.08 | 0.84 |
| agpat4_2 | 0.90 | 0.04 | 0.32 | 0.66 | 0.63 | 0.02 | 0.88 | <0.01 | 0.25 | 0.51 | 0.26 | 0.46 |
| agpat5_1 | 0.53 | 0.16 | 0.57 | 0.13 | -0.14 | 0.81 | -0.20 | 0.66 | 0.25 | 0.31 | 0.15 | 0.58 |
| agpat5_2 | 0.58 | 0.19 | 0.09 | 0.91 | 0.06 | 0.90 | -0.05 | 0.90 | 0.18 | 0.71 | 0.31 | 0.48 |
| apoa1_1 | 0.82 | 0.64 | 1.52 | 0.37 | 2.39 | <0.01 | 3.33 | <0.01 | 2.78 | 0.05 | 3.05 | 0.03 |
| apoa1_2 | 1.21 | 0.38 | 0.11 | 0.97 | 0.54 | 0.36 | 2.30 | <0.01 | -1.52 | 0.12 | -0.29 | 0.81 |
| apoa4a_1 | 1.85 | 0.60 | 1.40 | 0.66 | 2.92 | <0.01 | 5.35 | <0.01 | 0.14 | 0.95 | 1.04 | 0.51 |
| apoa4b_1 | -0.26 | 0.94 | 0.57 | 0.85 | 0.44 | 0.44 | 1.87 | <0.01 | 0.63 | 0.43 | 1.43 | 0.03 |
| apoa4b_2 | 0.14 | 0.90 | 0.16 | 0.90 | 1.52 | <0.01 | 2.97 | <0.01 | 0.84 | 0.29 | 1.18 | 0.09 |
| apoa4a_2 | 2.04 | 0.75 | 2.85 | 0.44 | 0.88 | 0.09 | 3.20 | <0.01 | -0.19 | 0.88 | 0.64 | 0.52 |
| apoa4c_1 | 1.59 | 0.54 | 2.71 | 0.18 | 3.50 | <0.01 | 5.00 | <0.01 | 0.44 | 0.76 | 0.84 | 0.47 |
| apoa4c_2 | 1.71 | 0.44 | 2.56 | 0.17 | 0.80 | 0.05 | 1.65 | <0.01 | 1.71 | 0.05 | 2.07 | <0.01 |
| apoba | 0.39 | 0.88 | -1.24 | 0.69 | -3.07 | <0.01 | -1.20 | 0.24 | -7.33 | <0.01 | -6.37 | <0.01 |
| apobb | 1.05 | 0.20 | 1.24 | 0.12 | 1.03 | 0.01 | 1.56 | <0.01 | 2.44 | 0.01 | 2.61 | <0.01 |
| apobc | 0.73 | 0.70 | -0.68 | 0.83 | -2.86 | <0.01 | -2.18 | <0.01 | -7.11 | <0.01 | -6.42 | <0.01 |
| cd36_1 | 1.83 | 0.20 | 2.20 | 0.09 | 3.04 | <0.01 | 2.50 | <0.01 | 6.50 | <0.01 | 5.91 | <0.01 |
| cd36_2 | 0.75 | 0.56 | 1.53 | 0.25 | 0.45 | 0.16 | 0.78 | <0.01 | 3.18 | <0.01 | 2.67 | <0.01 |
| cdipt | 0.27 | 0.38 | 0.28 | 0.42 | 0.26 | 0.23 | 0.36 | 0.07 | 0.10 | 0.61 | 0.07 | 0.73 |
| cds1a | -1.49 | 0.28 | -1.07 | 0.47 | -0.22 | 0.84 | -0.82 | 0.23 | 0.31 | 0.76 | -0.24 | 0.80 |
| cds1b | -0.05 | 0.95 | -0.49 | 0.35 | 0.36 | 0.16 | 0.46 | 0.05 | 0.20 | 0.42 | 0.04 | 0.88 |
| cds2_1 | 0.22 | 0.75 | 0.33 | 0.66 | -0.17 | 0.64 | -0.53 | 0.07 | -0.21 | 0.55 | -0.47 | 0.13 |
| cds2_2 | -0.39 | 0.38 | -0.17 | 0.79 | -0.63 | 0.01 | -0.82 | <0.01 | -0.53 | 0.02 | -0.80 | <0.01 |
| cept1a_1 | 0.01 | 0.99 | 0.01 | 0.99 | 0.72 | <0.01 | 0.71 | <0.01 | 0.78 | <0.01 | 0.96 | <0.01 |
| cept1a_2 | 0.08 | 0.84 | 0.06 | 0.91 | 0.41 | 0.13 | 0.17 | 0.55 | -0.11 | 0.75 | 0.05 | 0.87 |
| cept1b_1 | -0.14 | 0.75 | -0.07 | 0.92 | 0.14 | 0.68 | 0.27 | 0.34 | 0.14 | 0.56 | 0.04 | 0.88 |
| cept1b_2 | -0.09 | 0.83 | -0.31 | 0.45 | 0.14 | 0.59 | -0.15 | 0.51 | 0.19 | 0.42 | 0.21 | 0.34 |
| chka_1 | 1.91 | 0.06 | 1.91 | 0.07 | -0.49 | 0.45 | 0.60 | 0.30 | -1.55 | 0.14 | -2.12 | 0.10 |
| chka_2 | -0.19 | 0.83 | -0.10 | 0.93 | -1.72 | <0.01 | -0.70 | 0.07 | -1.00 | 0.01 | -0.62 | 0.14 |
| chkb | -0.12 | 0.80 | -0.01 | 0.99 | 1.23 | <0.01 | 1.97 | <0.01 | 0.62 | 0.05 | 0.59 | 0.05 |
| chpt1_1 | 0.36 | 0.29 | 0.16 | 0.75 | 0.83 | <0.01 | 1.31 | <0.01 | 0.87 | <0.01 | 0.96 | <0.01 |
| chpt1_2 | -0.64 | 0.07 | -0.45 | 0.26 | 0.31 | 0.21 | 0.72 | <0.01 | 0.34 | 0.14 | 0.22 | 0.35 |
| crls1 | 0.28 | 0.57 | 0.29 | 0.65 | -0.04 | 0.92 | 0.30 | 0.28 | 0.60 | <0.01 | 0.56 | 0.01 |
| ept1_1 | 1.28 | 0.05 | 0.33 | 0.77 | 0.96 | <0.01 | 1.28 | <0.01 | 0.86 | <0.01 | 0.92 | <0.01 |
| ept1_2 | 1.16 | 0.04 | 0.67 | 0.37 | 0.78 | 0.03 | 1.15 | <0.01 | 0.72 | <0.01 | 0.98 | <0.01 |
| etnk1_1 | 0.42 | 0.52 | 0.56 | 0.45 | 0.19 | 0.45 | -0.16 | 0.51 | -0.38 | 0.08 | -0.32 | 0.14 |
| etnk1_2 | -0.29 | 0.34 | -0.16 | 0.70 | -0.16 | 0.51 | -0.45 | 0.03 | -1.29 | <0.01 | -1.38 | <0.01 |
| etnk2_1 | 1.79 | <0.01 | 0.83 | 0.39 | -0.10 | 0.93 | 0.27 | 0.75 | 0.10 | 0.86 | 0.19 | 0.70 |
| etnk2_2 | 0.81 | 0.05 | 0.40 | 0.50 | 1.90 | <0.01 | 2.78 | <0.01 | 2.67 | <0.01 | 2.80 | <0.01 |
| fabp1 | -0.02 | 0.99 | 1.86 | 0.14 | 0.30 | 0.57 | 1.78 | <0.01 | -0.39 | 0.69 | -0.28 | 0.76 |
| gpat1 | 0.57 | 0.05 | 0.25 | 0.54 | 0.39 | 0.15 | 0.71 | <0.01 | 1.11 | 0.01 | 1.19 | <0.01 |
| gpat2 | 0.04 | 0.95 | -0.08 | 0.91 | -0.36 | 0.12 | -0.53 | 0.01 | -0.94 | <0.01 | -1.02 | <0.01 |
| gpat3a_1 | 0.88 | 0.06 | 0.29 | 0.71 | -0.25 | 0.63 | -0.60 | 0.15 | -0.04 | 0.92 | -0.13 | 0.72 |
| gpat3a_2 | -0.18 | 0.78 | -0.26 | 0.73 | -0.30 | 0.51 | -0.48 | 0.25 | -0.39 | 0.15 | -0.31 | 0.27 |
| gpat3b_1 | 0.55 | 0.29 | 0.54 | 0.37 | 0.38 | 0.12 | 0.84 | <0.01 | -0.37 | 0.15 | -0.25 | 0.32 |
| gpat3b_2 | 1.42 | 0.02 | 1.39 | 0.02 | 0.29 | 0.26 | 0.64 | <0.01 | 0.44 | 0.12 | 0.25 | 0.40 |
| gpat4b_1 | -0.62 | 0.06 | -0.81 | <0.01 | -0.39 | 0.28 | -0.97 | <0.01 | -0.58 | 0.09 | -0.73 | 0.03 |
| gpat4b_2 | -0.13 | 0.83 | -0.19 | 0.79 | -0.50 | 0.27 | -0.97 | 0.02 | -0.46 | 0.19 | -0.60 | 0.07 |
| lclat1 | 0.10 | 0.82 | 0.07 | 0.90 | -0.19 | 0.33 | -0.05 | 0.81 | 0.00 | 0.99 | -0.03 | 0.90 |
| lpcat1a | -0.63 | 0.23 | -0.05 | 0.95 | -0.10 | 0.83 | -0.35 | 0.27 | -0.64 | <0.01 | -0.52 | 0.01 |
| lpcat2_1 | -0.43 | 0.43 | -0.19 | 0.81 | -0.12 | 0.73 | -0.48 | 0.05 | -0.25 | 0.33 | -0.48 | 0.03 |
| lpcat2_2 | -0.42 | 0.37 | -0.15 | 0.83 | -0.19 | 0.75 | -0.50 | 0.28 | 0.12 | 0.72 | 0.03 | 0.93 |
| lpcat3_1 | 1.00 | 0.03 | 0.11 | 0.91 | 0.82 | <0.01 | 1.40 | <0.01 | 1.31 | <0.01 | 1.07 | 0.02 |
| lpcat3_2 | 0.56 | 0.25 | 0.05 | 0.95 | 0.74 | <0.01 | 1.45 | <0.01 | 0.53 | 0.03 | 0.56 | 0.02 |
| lpcat4_1 | 0.32 | 0.40 | 0.38 | 0.34 | 0.02 | 0.94 | 0.20 | 0.35 | -0.08 | 0.78 | 0.12 | 0.65 |
| lpgat1_1 | 0.49 | 0.33 | 0.12 | 0.89 | 1.35 | <0.01 | 1.91 | <0.01 | 0.87 | <0.01 | 1.04 | <0.01 |
| lpgat1_2 | -0.07 | 0.97 | -0.43 | 0.77 | 0.81 | <0.01 | 1.17 | <0.01 | 0.81 | <0.01 | 0.87 | <0.01 |
| lpiat1 | 0.29 | 0.45 | 0.13 | 0.82 | 0.71 | <0.01 | 0.85 | <0.01 | 0.44 | <0.01 | 0.41 | <0.01 |
| lpin1_1 | 0.31 | 0.85 | -0.39 | 0.85 | -1.40 | 0.11 | -1.29 | 0.10 | -1.01 | 0.14 | -1.21 | 0.10 |
| lpin1_2 | -0.70 | 0.19 | -1.19 | 0.01 | -1.40 | <0.01 | -2.36 | <0.01 | -0.78 | 0.02 | -1.08 | <0.01 |
| lpin2_1 | 0.41 | 0.56 | 0.43 | 0.62 | 0.76 | <0.01 | 0.98 | <0.01 | 0.77 | 0.10 | 0.83 | 0.05 |
| lpin2_2 | -0.77 | 0.05 | -0.56 | 0.23 | 0.02 | 0.94 | 0.14 | 0.58 | -0.12 | 0.76 | 0.09 | 0.79 |
| lpin3_1 | 0.33 | 0.69 | -0.69 | 0.38 | -0.23 | 0.66 | -0.21 | 0.65 | -0.31 | 0.40 | -0.65 | 0.05 |
| lpin3_2 | 0.40 | 0.22 | -0.01 | 0.99 | 1.16 | <0.01 | 1.44 | <0.01 | 0.39 | 0.03 | 0.30 | 0.10 |
| mboat2a_1 | 0.03 | 0.95 | -0.05 | 0.94 | -1.12 | <0.01 | -1.08 | <0.01 | -0.48 | 0.08 | -0.37 | 0.19 |
| mboat2a_2 | 0.33 | 0.52 | 0.65 | 0.15 | -1.66 | 0.05 | -0.58 | 0.52 | 0.13 | 0.81 | 0.55 | 0.18 |
| mboat2b_1 | -0.13 | 0.89 | 0.23 | 0.82 | 0.16 | 0.81 | 0.10 | 0.86 | 0.27 | 0.58 | 0.08 | 0.88 |
| mboat2b_2 | -0.85 | 0.04 | 0.02 | 0.98 | -1.43 | <0.01 | -1.66 | <0.01 | -1.09 | <0.01 | -1.27 | <0.01 |
| mtp_1 | -0.04 | 0.96 | -0.17 | 0.83 | 0.70 | 0.03 | 1.14 | <0.01 | 1.02 | 0.02 | 1.24 | <0.01 |
| mtp_2 | 0.86 | 0.67 | 1.76 | 0.35 | 0.14 | 0.71 | 0.95 | <0.01 | 2.05 | 0.09 | 2.10 | 0.08 |
| pcyt1aa | 0.50 | 0.16 | 0.29 | 0.55 | 0.32 | 0.27 | 0.09 | 0.78 | 0.05 | 0.86 | -0.02 | 0.96 |
| pcyt1ab_1 | 0.93 | 0.04 | 0.61 | 0.28 | 1.15 | <0.01 | 1.63 | <0.01 | 1.05 | <0.01 | 1.31 | <0.01 |
| pcyt1ab_2 | -0.30 | 0.31 | -0.18 | 0.65 | 0.01 | 0.97 | 0.06 | 0.79 | -0.07 | 0.77 | 0.00 | 1.00 |
| pcyt1ba_1 | 0.58 | 0.19 | 0.69 | 0.12 | 0.45 | 0.47 | 0.25 | 0.68 | 1.02 | 0.03 | 0.93 | 0.06 |
| pcyt1bb_1 | 2.16 | 0.38 | 3.96 | 0.09 | 0.32 | 0.30 | 1.27 | <0.01 | 0.54 | 0.52 | 0.90 | 0.23 |
| pcyt1bb_2 | 0.40 | 0.78 | 0.87 | 0.50 | 0.29 | 0.32 | 0.94 | <0.01 | 0.70 | 0.20 | 0.97 | 0.05 |
| pcyt2a | 0.23 | 0.61 | -0.52 | 0.22 | 0.11 | 0.77 | 0.68 | 0.01 | -0.18 | 0.47 | 0.04 | 0.89 |
| pcyt2b | 0.35 | 0.84 | 1.01 | 0.47 | 0.38 | 0.61 | 2.47 | <0.01 | 1.26 | <0.01 | 1.32 | <0.01 |
| pcyt2c_1 | 0.96 | 0.57 | 1.52 | 0.33 | 2.57 | <0.01 | 4.64 | <0.01 | 1.92 | <0.01 | 2.60 | <0.01 |
| pcyt2c_2 | 1.34 | 0.28 | 1.57 | 0.18 | 2.46 | <0.01 | 4.17 | <0.01 | 2.90 | <0.01 | 3.68 | <0.01 |
| pemt | 0.21 | 0.69 | 0.21 | 0.75 | -0.14 | 0.67 | 0.18 | 0.52 | -0.11 | 0.74 | -0.21 | 0.44 |
| pgs1_1 | -0.56 | 0.14 | -0.55 | 0.18 | -0.93 | <0.01 | -0.63 | <0.01 | -0.08 | 0.79 | -0.23 | 0.35 |
| pgs1_2 | -0.55 | 0.18 | -0.76 | 0.06 | -0.43 | 0.15 | -0.51 | 0.07 | -0.13 | 0.68 | -0.27 | 0.36 |
| pisd_1 | 0.00 | 1.00 | 0.11 | 0.84 | -0.34 | 0.15 | -0.04 | 0.88 | 0.12 | 0.61 | 0.25 | 0.24 |
| pisd_2 | -0.34 | 0.33 | -0.70 | 0.02 | -0.51 | 0.13 | -0.33 | 0.32 | -1.02 | <0.01 | -0.93 | <0.01 |
| plpp1a_1 | 1.91 | 0.01 | 1.92 | 0.01 | 1.53 | 0.03 | -0.24 | 0.77 | 1.81 | <0.01 | 1.09 | 0.13 |
| plpp1a_2 | 2.65 | <0.01 | 2.22 | <0.01 | 3.65 | <0.01 | 3.23 | <0.01 | 4.09 | <0.01 | 3.92 | <0.01 |
| plpp1b_1 | 0.49 | 0.40 | 0.51 | 0.43 | 0.27 | 0.20 | 0.41 | 0.03 | 0.20 | 0.38 | 0.20 | 0.37 |
| plpp1b_2 | 0.18 | 0.66 | 0.23 | 0.63 | 0.08 | 0.78 | 0.23 | 0.32 | -0.02 | 0.95 | -0.07 | 0.81 |
| plpp2a_1 | 0.54 | 0.45 | -0.30 | 0.77 | -0.58 | 0.12 | -0.49 | 0.18 | -0.27 | 0.70 | -0.58 | 0.37 |
| plpp2a_2 | 0.21 | 0.83 | -0.36 | 0.74 | 0.56 | 0.16 | 0.05 | 0.92 | 0.28 | 0.33 | 0.30 | 0.26 |
| plpp2b_1 | 0.57 | 0.37 | 0.32 | 0.72 | 0.26 | 0.43 | 0.37 | 0.19 | 0.42 | 0.06 | 0.34 | 0.12 |
| plpp2b_2 | -0.37 | 0.65 | 0.19 | 0.88 | 0.31 | 0.56 | 0.74 | 0.10 | -0.39 | 0.46 | -0.10 | 0.87 |
| plpp3a_1 | 0.07 | 0.96 | 0.00 | 1.00 | 0.16 | 0.80 | -0.31 | 0.53 | 0.32 | 0.64 | 0.42 | 0.53 |
| plpp3a_2 | 0.52 | 0.40 | 0.45 | 0.57 | -0.21 | 0.55 | -0.48 | 0.10 | -0.98 | <0.01 | -0.94 | <0.01 |
| plpp3b_1 | -0.64 | 0.22 | -0.28 | 0.70 | -0.41 | 0.27 | -0.38 | 0.28 | 0.24 | 0.58 | 0.15 | 0.73 |
| plpp3b_2 | -0.14 | 0.83 | 0.46 | 0.38 | -0.61 | 0.11 | -0.87 | 0.01 | 0.00 | 1.00 | -0.18 | 0.69 |
| plpp3d | 0.89 | <0.01 | 0.91 | <0.01 | -0.12 | 0.67 | -0.02 | 0.94 | 0.21 | 0.34 | 0.06 | 0.81 |
| pmt_1 | 3.06 | <0.01 | 0.80 | 0.58 | 1.21 | 0.62 | 0.70 | 0.71 | -1.62 | 0.41 | -2.07 | 0.29 |
| pmt_2 | 3.01 | <0.01 | 2.58 | <0.01 | -2.04 | 0.32 | -0.90 | 0.52 | -5.90 | <0.01 | -5.87 | <0.01 |
| pss | -1.50 | 0.07 | -1.08 | 0.24 | -2.67 | <0.01 | -2.16 | 0.01 | -1.75 | <0.01 | -1.16 | 0.08 |
| ptdss1a | -0.39 | 0.53 | -0.34 | 0.66 | 0.43 | 0.51 | -0.04 | 0.96 | -0.12 | 0.80 | -0.65 | 0.08 |
| ptdss1b | 0.55 | 0.42 | -0.18 | 0.87 | 0.47 | 0.20 | 0.33 | 0.36 | -0.27 | 0.43 | -0.37 | 0.24 |
| ptdss1c_1 | 0.76 | 0.24 | 0.33 | 0.73 | -0.55 | 0.16 | -0.38 | 0.33 | -0.04 | 0.93 | -0.22 | 0.57 |
| ptdss1c_2 | -1.56 | 0.03 | -1.52 | 0.02 | -1.00 | 0.02 | -0.98 | 0.02 | -0.36 | 0.60 | -1.04 | 0.09 |
| ptdss2 | 0.41 | 0.70 | -0.18 | 0.91 | -0.34 | 0.37 | -0.09 | 0.82 | 0.05 | 0.92 | 0.21 | 0.64 |
| sar1a_2 | 0.11 | 0.89 | 0.14 | 0.89 | 0.87 | <0.01 | 1.30 | <0.01 | 0.90 | <0.01 | 0.80 | <0.01 |
| sar1ba_1 | 0.65 | 0.03 | 0.39 | 0.30 | 1.04 | <0.01 | 1.56 | <0.01 | 0.85 | <0.01 | 0.84 | <0.01 |
| sar1ba_2 | 0.63 | 0.05 | 0.33 | 0.45 | 0.83 | <0.01 | 1.23 | <0.01 | 0.85 | <0.01 | 0.84 | <0.01 |
| sar1bb_1 | 0.55 | 0.25 | 0.45 | 0.45 | 0.70 | <0.01 | 1.11 | <0.01 | 0.20 | 0.51 | 0.43 | 0.10 |
| sar1bb_2 | 0.68 | 0.34 | 0.38 | 0.71 | 0.86 | <0.01 | 1.48 | <0.01 | 0.40 | 0.09 | 0.58 | <0.01 |
| taz_1 | -0.05 | 0.95 | -0.44 | 0.43 | -0.18 | 0.61 | 0.02 | 0.95 | -0.06 | 0.90 | -0.30 | 0.44 |
| taz_2 | -0.34 | 0.59 | -0.41 | 0.59 | -0.33 | 0.27 | -0.30 | 0.29 | -0.53 | 0.06 | -0.57 | 0.04 |
